# Metabolites Discovery from *Streptomyces xanthus*: Exploring the Potential of Desert Microorganisms

**DOI:** 10.3390/biology14020164

**Published:** 2025-02-06

**Authors:** Xinrong Luo, Zhanwen Liu, Zhanfeng Xia, Xiaoxia Luo, Juan Zhang, Ailiang Chen, Haoxin Wang, Chuanxing Wan, Lili Zhang

**Affiliations:** 1 State Key Laboratory Incubation Base for Conservation and Utilization of Bio-Resource in Tarim Basin, College of Life Science and Technology, Tarim University, Alar 843300, China; luoxinrong365@163.com (X.L.); zwzky@163.com (Z.L.); fenge3721@163.com (Z.X.); xxluo415@163.com (X.L.); wanchuanxing@163.com (C.W.); 2 Key Laboratory of Agro-Product Quality and Safety, Institute of Quality Standard & Testing Technology for Agro-Products, Chinese Academy of Agricultural Sciences, Beijing 100081, China; 15110679191@yeah.net (J.Z.); chenailiang@caas.cn (A.C.); 3 State Key Laboratory of Microbial Technology, Shandong University, Qingdao 266237, China; wanghaoxin@sdu.edu.cn

**Keywords:** actinomycete, polyphasic taxonomy, genomics, metabolomics, thiolutin, Taklamakan desert

## Abstract

The Taklamakan Desert is an extreme environment supporting a unique and diverse microbial community with significant potential for exploration. Strain TRM70308^T^, isolated from desert soil, shares 98.43% 16S rRNA gene sequence similarity with *Streptomyces alkaliterrae* OF1^T^. Polyphasic taxonomy confirmed TRM70308^T^ as a novel species, named *Streptomyces xanthus*. Genomic analysis revealed that only one of the strain’s 25 biosynthetic gene clusters (BGCs) formed a cluster of gene families (CGFs) within the MIBiG database, emphasizing its genomics uniqueness. LC-MS/MS and Feature-Based Molecular Networking (FBMN) identified 33 metabolites across various categories, including alkaloids, saponins, benzoic acids, and benzofurans, most of which remain uncharacterized. Further chemical investigation led to the isolation of one novel compound, aconicarpyrazine C, and four known compounds: thiolutin, dibutyl phthalate, bis(2-ethylhexyl) phthalate, and *N*-acetyltryptamine. Thiolutin exhibited strong activity against five local fungal pathogens that cause plant diseases, with a production yield of 270 mg/L. These results establish a foundation for pilot-scale thiolutin production and its potential development as an antifungal agent for agricultural applications. Our findings highlight deserts as a valuable source of novel actinomycetes and bioactive natural products with immense potential for future research and development.

## Simple Summary

The Taklamakan Desert is a harsh and extreme environment, yet its microorganisms hold untapped potential to benefit agriculture and medicine. In this study, we discovered a new microbial species, *Streptomyces xanthus*, isolated from desert soil. Genomic and metabolomic analyses revealed that this microorganism produces several unknown compounds. We isolated one novel and four known compounds from this strain, all reported for the first time in desert environments. Notably, one known compound, thiolutin, showed strong activity against five local plant pathogenic fungi, with a production yield of 270 mg/L. These findings highlight the desert as a reservoir of new microbial species, holding great potential for discovering important bioactive compounds.

## 1. Introduction

The Taklamakan Desert is the second most mobile desert in the world, and is characterized by intense solar radiation, significant diurnal temperature fluctuations, prolonged periods of drought and heat, nutrient scarcity, and soil salinization. The microbial community has evolved with a greater degree of complexity in its adaptive mechanisms and a higher level of diversity in this extreme environment [1]. Microorganisms continuously adapt and evolve in this environment to develop unique metabolic abilities. Li Shuai [2] recently identified numerous novel bacteria in desert soil using culturomics-based metagenomics. Ting Wang’s research [1], which was conducted in the Taklamakan Desert, led to the discovery of 320 strains of actinomycetes, which were distributed across 23 genera and included 16 potential new species. Notably, strains from 20 of these genera exhibited activity against at least one pathogen. The continuous discovery of new compounds and genes from desert actinomycetes highlights the role of deserts as repositories of microbial diversity and novel genetic material [3,4,5]. Despite recent reports on desert microorganisms [6], the Taklamakan Desert remains largely unexplored, emphasizing the need for further exploration to harness its potential and contribute to the efforts to address antibiotic resistance.

For many years, natural products from microorganisms, particularly actinomycetes, have been a rich source of bioactive compounds [7]. Renowned for their extensive secondary metabolic capabilities, these microorganisms produce more than two-thirds of the antibiotics currently in clinical use [8]. In 1943, Waksman and Henrici first described the genus *Streptomyces* and isolated the antibiotic streptomycin [9], marking a new era in antibiotic research and highlighting *Streptomyces* species for their robust metabolic capacity. The genomes of *Streptomyces* are vast and intricate, and harbor numerous biosynthetic gene clusters [10]. Antibiotics produced by *Streptomyces*, such as erythromycin [11], tetracycline [12], avermectin [13], and validamycin [14], have provided significant resources and drug candidates for the pharmaceutical, agricultural, and industrial sectors. Furthermore, Wang isolated a novel antibiotic from the streptogramin family, named desulfurizing griseo-viridin, from a strain of *Streptomyces* discovered in the Taklamakan Desert. Additionally, a representative member of the established polyunsaturated macrolactones family, griseoviridin, was also identified, and both compounds exhibited antimicrobial activity through the inhibition of translation [1]. However, despite these achievements, the discovery of new exploitable antibiotics remains a significant challenge owing to the growing prevalence of antibiotic resistance and other application issues.

In this study, we isolated an actinomycete strain from the desert and identified it as a novel species, named *Streptomyces xanthus*, using polyphasic taxonomic methods. To further explore the biosynthetic potential of this strain, we conducted comprehensive genomics and Feature-Based Molecular Networking (FBMN) metabolomic analyses. The results revealed that the strain exhibits not only genomic uniqueness but also significant diversity in its metabolic products. The crude extracts of this strain exhibited significant activity against fungi, gram-positive bacteria, and gram-negative bacteria. Consequently, we proceeded to isolate and purify the bioactive substances, ultimately identifying a novel pyrazine compound, along with four known compounds. We further evaluated their activity against five local plant diseases.

## 2. Materials and Methods

### 2.1. Isolation and Maintenance of the Microorganism

Strain TRM70308^T^ was isolated from soil samples collected in the Taklamakan Desert, Xinjiang, China, and the samples were stored at 4 °C prior to cultivation. To prepare the samples for isolation, they were dried in a hot-air oven at 60 °C for 2 h. From the pre-treated soil samples, 1 g was weighed, dissolved in 9 mL of 0.9% saline, and transferred to a 50 mL triangular flask. The solution was then incubated at room temperature (120 rpm) for 30 min. The solution was serially diluted with sterile distilled water (10, 100, 1000, and 10,000 times) and spread on isolation media, which were incubated at 37 °C for 30 days. The isolation media consisted of soluble starch 20 g, KNO_3_ 1 g, K_2_HPO_4_ 0.5 g, MgSO_4_·7H_2_O 0.5 g, NaCl 1 g, FeSO_4_·7H_2_O 0.01 g, CaCO_3_ 1 g, agar 18 g, soil extract 1000 mL, pH 8.0, and supplemented with nalidixic acid (50 mg/L) and anti-fungal (100 mg/L) to inhibit the growth of most bacteria and fungi. The isolates were purified using International Actinomycete Project (ISP-4) medium, and the purified strains were stored in 20% glycerol and freeze-dried skim milk.

### 2.2. Molecular Characterization

DNA was isolated using an E.Z.N.A.^®^ Tissue DNA Kit (Omega Bio-Tek, Guangzhou, China). PCR amplification of 16S rRNA gene sequences was performed according to the method described by Nicole [15]. PCR products were purified using the E.Z.N.A.^®^ Gel Extraction Kit (Omega Bio-Tek) and sent to Sangon Biotech (Shanghai, China) for sequencing in both directions. The 16S rRNA gene sequence similarity was calculated for related type strains using the EzBioCloud web server [16], and subsequently submitted to the GenBank database.

Genome sequencing was conducted by Personal Biotechnology (Shanghai, China) using the Nanopore PromethION48 (New York, NY, USA) and Illumina NovaSeq platforms (San Diego, CA, USA). A5-MiSeq [17] and SPAdes [18] were used for ab initio assembly, and base correction was performed using Pilon to obtain the final genomic sketch. DNA sequences of 89 housekeeping genes were extracted and MLSA was conducted using autoMLST (https://automlst.ziemertlab.com, accessed on 3 December 2024) [19]. Phylogenetic trees were constructed using the NJ, ML, and ME methods using MEGA-X software [20], with *Kitasatospora setae* KM-6054^T^ as an outgroup. Bootstrap replications (1000 replicates) were performed. The ANI was determined using OrthoANI 0.93.1 [21]. The phylogenomic tree of the whole-genome sequences was constructed using the type (strain) Genome Server platform (https://tygs.dsmz.de/, accessed on 2 December 2024.) [22].

### 2.3. Physiological and Chemotaxonomic Characterization

The cultural characteristics of the strain TRM 70308^T^ were observed with confidence on ISP 1–7 media after exactly two weeks of incubation at 37 °C [23]. The colors of the aerial mycelia, substrate mycelia, and spores were assessed by comparison with ISCC-NBS color charts [24]. Mycelial and spore morphologies were observed by light microscopy (OLYMPUS CX23, Tokyo, Japan) and scanning electron microscopy (Quanta; FEI, Hillsboro, OR, USA) after incubation on ISP4 plates at 37 °C for 7 days. The strains were tested for growth at different temperatures, pH values, and NaCl tolerances on Gauze’s medium after 14 days of incubation. Sole carbon utilization, cellulose degradation, and starch and nitrate reduction were investigated as described previously [23]. The sugar composition and isomers of diaminopimelic acid, polar lipids, fatty acids, and menaquinones in the whole cell were analyzed using established methods [25,26,27].

### 2.4. BGCs Mining and Network Analysis

BGCs were identified using antiSMASH (v7.1.0) online [28]. BiG-SCAPE (v1.1.5) was executed in auto mode, and experimentally validated BGCs from the MIBiG database 3.0 served as a reference [29]. The protein sequences predicted by antiSMASH for each BGC were searched within the predicted Pfam domains, and a similarity network was constructed using a Jaccard index of 0.3 to classify the diverse BGCs into GCFs. The resulting clustering network graphs were visualized and analyzed using Cytoscape 3.10.0 [30].

### 2.5. Metabolites Analysis Using LC-MS/MS and GNPS

A single colony of the TRM70308^T^ strain was inoculated into 50 mL of tryptic soy broth (TSB) medium in a 250 mL flask and incubated at 120 rpm and 37 °C for 48 h to obtain the seed culture. Then, 2 mL of the seed culture was transferred into a 500 mL Erlenmeyer flask containing 150 mL of ISP3 medium and fermented at 120 rpm and 37 °C for 5 days. The fermentation broth was filtered to remove the bacterial cells, and 10 mL of the broth was concentrated under reduced pressure. It was then dissolved sequentially in 1 mL each of methanol and water and adjusted to a final volume of 500 μL in methanol solution. Liquid chromatography-tandem mass spectrometry (LC-MS/MS) was conducted on an AB SCIEX TripleTOF 6600 system with the following ESI conditions: capillary voltage at 2500 V, source temperature at 120 °C, and dissociation temperature at 350 °C. Detection was performed in positive ion mode using MSE acquisition.

The raw LC-MS/MS data were converted to mzML format, and the MS2 spectra were analyzed using MSDIAL with parameters: sigma window value of 0.1, MS/MS abundance cutoff of 5, MS1 tolerance of 0.01, and MS2 tolerance of 0.0025, with default values for other parameters. The MS2 spectra, exported in mgf format, were analyzed on the FBMN platform within the GNPS ecosystem. Molecular networks were generated using the default parameters of the FBMN workflow. Further analysis and refinement of these networks were performed using Cytoscape 3.10.0 [30].

### 2.6. Preliminary Assessment of Activity

The method for obtaining crude extracts from the strain is consistent with the procedure described in Section 2.5. The activity of the crude extract was initially assessed using the filter paper agar diffusion method. Specifically, 20 μL of both methanol and aqueous- phase crude extracts were added onto a 6 mm diameter sterile filter paper disk. After methanol evaporation and drying, the disks were placed on media inoculated with 100 μL each of *Candida albicans* (ATCC 64550), *Escherichia coli* (ATCC 25922), and *Staphylococcus aureus* (ATCC 6538). Methanol was used as the blank control. Inhibition tests were conducted in triplicate, and the average diameter of the inhibition zones was calculated.

### 2.7. Isolation and Yield Determination of Compound

A single colony of the TRM70308^T^ strain was inoculated into 50 mL of TSB medium in a 250 mL flask and incubated at 120 rpm at 37 °C for 48 h to obtain the seed culture. Then, 2 mL was inoculated into a 500 mL triangular flask containing 150 mL ISP3 medium, and fermented at 120 rpm and 37 °C for 5 days, with a total volume of 15 L. The upper layer of biomass was adsorbed onto D101 macroporous resin and subsequently eluted with pure water and 100% methanol. After concentrating the methanol eluate under reduced pressure and washing it three times with a small amount of methanol, a yellow solid insoluble in methanol was obtained, identified as compound **12**. The methanol-soluble fraction was further eluted with methanol on a Sephadex LH-20 column, resulting in four subfractions (S1–S4). Subfraction S2 underwent HPLC analysis using a ZORBAX Eclipse XDB-C18 column (250 × 9.4 mm, 5 µm, 4.0 mL/min) and was further purified by HPLC, eluting with 70% methanol/water to obtain compound **35**. Subfraction S3 was purified by HPLC and eluted with 45% methanol/water to yield compounds **21** and **34**. Subfraction S4 was purified by HPLC and eluted with 60% methanol/water to obtain compound **36**. NMR spectra were obtained using a 500 MHz NMR instrument (Bruker, Switzerland). ^1^H-NMR, ^13^C-NMR, HSQC, and HMBC NMR spectra of the compounds were analyzed to determine their structures.

The standard compounds were accurately weighed and diluted to the desired concentration using dimethyl sulfoxide. The standard solution was then further diluted to a series of concentrations. A Shim-pack GIST C18 column (4.6 mm × 25 cm, 5 µm) was used, with methanol and water as the mobile phases. The detection conditions were set with a methanol gradient from 10% to 100% over 0 to 40 min, at a flow rate of 1 mL/min. A 10 µL sample was injected into the column, and the peak areas of thiolutin at different concentrations were detected at 388 nm at 18.32 min. The standard curve was plotted using these peak areas and was utilized to analyze the yield of compounds in the crude extract of the strain.

### 2.8. Antibacterial Activity and MIC Assay

The MIC for the pathogens were determined using the Clinical and Laboratory Standards Institute method. Compounds were prepared at defined concentrations using DMSO and determined against *Verticillium dahliae* ACCC 36211, *F. oxysporum* ACCC 31038, *Valsa pyri* WTZ-19, *Erwinia amylovor* TRM-03, and *Alternaria gaisen* TRM-09. Amphotericin B was used as a reference drug, DMSO was used as a blank control, and blank medium was used as a negative control.

A volume of 100 µL of a prepared compound solution was added to the first well of a 96-well plate, and the drug was serially diluted 2-fold to the final well. In the first ten wells, 100 µL of each pathogen should be added at a concentration of approximately 2 × 10^6^ organisms/mL. while the 11th well contained only the blank medium, and the 12th well included the pathogen without thiolutin. After 48 h of incubation at 35 °C, the lowest concentration of each test compound at which a color change occurred was recorded as the primary MIC value.

## 3. Results

### 3.1. Morphological and Cultural Characteristics

**Figure 1:**
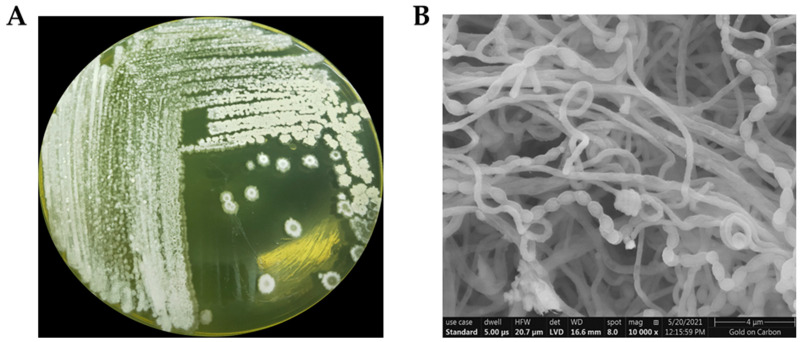
(**A**) Colony morphology of strain TRM70308^T^ after a five-day incubation at 37 °C in ISP4, and (**B**) scanning electron microscopy characteristics. The colonies were dry and radial, with unbranched mycelium. The spores were ovoid, smooth-surfaced, and arranged in curved chains.

Strain TRM70308^T^ was cultured in ISP4 medium at 37 °C for five days. During this period, yellow substrate mycelia were observed alongside white aerial mycelia on the surface. The colonies were flat and dry with filiform margins, displaying typical morphological characteristics of actinomycetes (Figure 1A). Scanning electron microscopy revealed that the mycelia were sparsely branched and lacked transverse septa. The abundant aerial mycelia formed spore chains of varying lengths. The spores were ovoid and featured smooth spineless surfaces, whereas the spore chains exhibited a spiral shape (Figure 1B).

### 3.2. Genomic Features and Phylogeny

**Figure 2:**
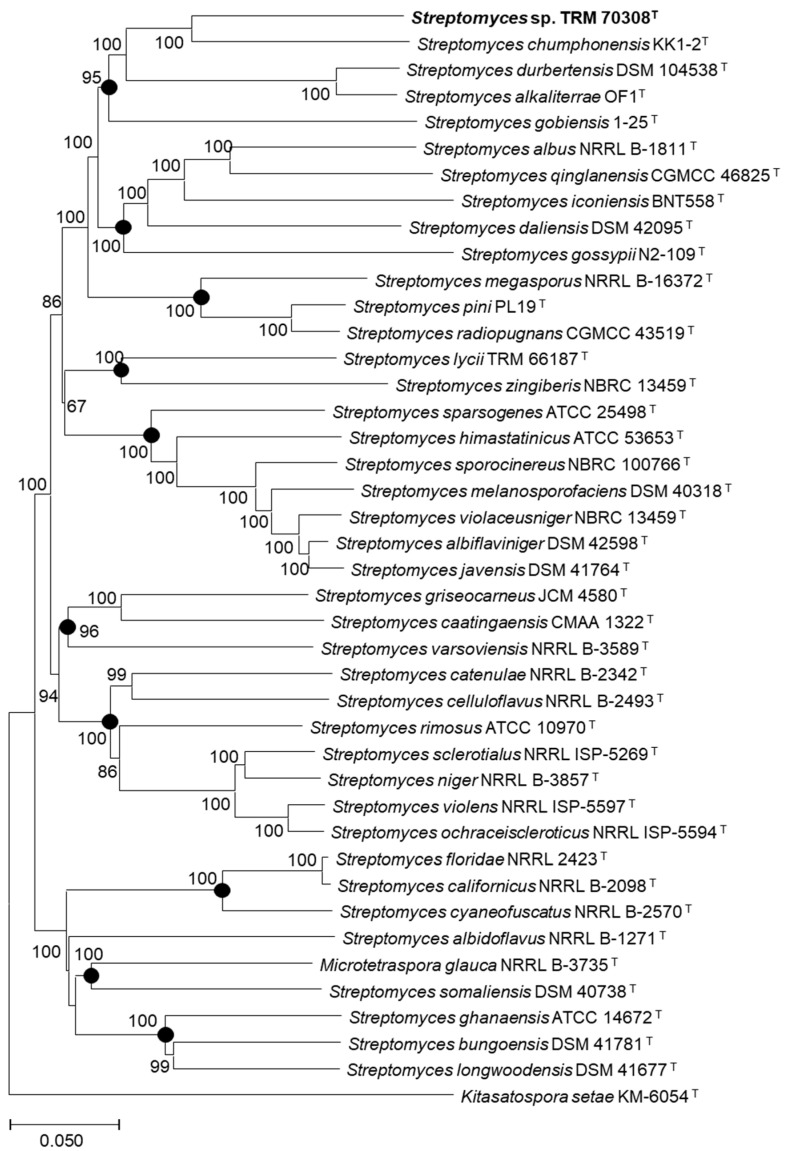
Phylogenetic tree of strain TRM70308^T^ and related strain based on MLSA (neighbor-joining method). Bootstrap percentages from 1000 replicates are indicated at the nodes, with only values greater than 50% displayed. The scale bar represents 0.05 substitutions per nucleotide position. The presence of black dots on a branch signifies that the branch also appears in Maximum Likelihood and Minimum Evolution method (T, type strains).

Sequence analysis of the 16S rRNA gene of strain TRM70308^T^ indicated that the similarity (98.43%) was observed between TRM70308^T^ and strains *Streptomyces alkaliterrae* OF1^T^ and *Streptomyces lycii* TRM 66187^T^, followed by *Streptomyces chumphonensis* KK1-2^T^ and *Streptomyces gobiensis* 1-25^T^ (98.15%). A multi-locus sequence analysis (MLSA) was conducted using 89 housekeeping genes (Figure 2). The results demonstrated that strain TRM70308^T^ formed a stable branch with strain *S. chumphonensis* KK1-2^T^, achieving a bootstrap value of 100%. Moreover, the MLSA distance significantly exceeded the species-level threshold of 0.007, and the evolutionary trees constructed using the ML and ME methods yielded consistent results (Figures S1 and S2).

The phylogenetic tree of 16S rRNA based on neighbor-joining (NJ) analysis revealed that strain TRM70308^T^ formed an independent branch within the *Streptomyces* genus, suggesting its potential for classification as a new species (Figure S3). The phylogenetic tree constructed using both the maximum likelihood (ML) and minimum evolution (ME) methods yielded identical results (Figures S4 and S5). However, the bootstrap values for the branches of the evolutionary tree constructed using the three methods were less than 50%, indicating branch instability.

**Figure 3:**
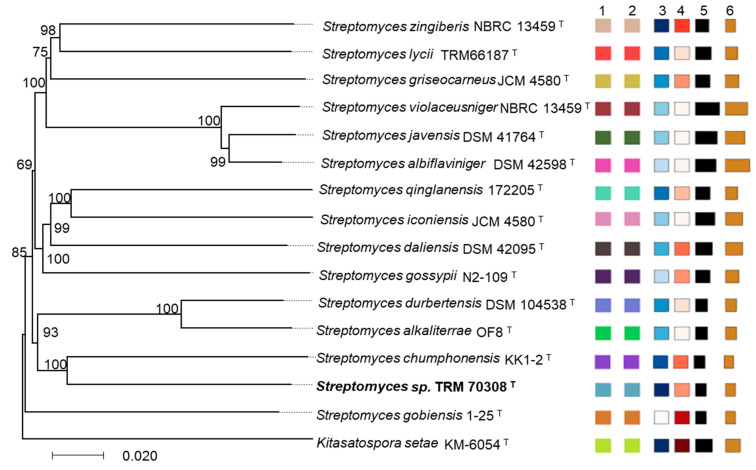
Evolutionary and Morphological Characteristics of Strain TRM70308^T^. Phylogenomic tree based on genome sequences from strain TRM70308^T^ and its related type strains using the Type Strain Genome Server (TYGS). The numbers above the branches represent genome blast distance phylogeny (GBDP) pseudo-bootstrap support values greater than 60% from 1000 replications. The tree was rooted at the midpoint. The positions of the strains of interest are indicated in bold. 1. Species cluster, 2. Subspecies cluster, 3. Percent G+C, 4. Delta statistics, 5. Genome size (in bp), 6. Protein count (the different colors represent the differences in indicator content between the strains).

The TRM70308^T^ genome has a size of 5.6 Mb, a (G+C) content of 74.5 mol%, and contains 4910 protein-coding genes. Whole-genome comparisons and phylogenomic analyses are recommended to provide greater taxonomic resolution when comparing potentially novel *Streptomyces* species with closely related taxa. In this study, a phylogenomic tree was generated, revealing that strain TRM70308^T^ was most closely related to *S. chumphonensis* KK1-2^T^, forming a distinct clade with high bootstrap support (100%). Although both strains evolved on the same branch, notable discrepancies were observed in genome size, protein content, number of gene clusters, and (G+C) mol%. In contrast, the remaining *Streptomyces* type strains, *S. alkaliterrae* OF1^T^, *S. lycii* TRM 66187^T^, and *S. gobiensis* 1-25^T^, were more distantly related to strain TRM70308^T^. These observations collectively supported the hypothesis that this strain represents a potentially novel species of *Streptomyces* (Figure 3). TRM70308^T^ exhibited an average nucleotide identity (ANI) of 81.89% with its closest related strain *S. chumphonensis* KK1-2^T^. Additionally, the ANI values for all other potentially related strains remained significantly below the 95–96% threshold for species delimitation (Figure S6).

### 3.3. Physiological and Chemical Characterization Results

Phenotypic differences were observed between TRM70308^T^ and its closely related phylogenetic neighbors, such as *S. chumphonensis* KK1-2^T^, S. *lycii* TRM66187^T^, *S. gobiensis* 1-25^T^, *S. alkaliterrae* OF1^T^_,_ and *S. durbertensis* NEAU-S1GS20^T^ (Table 1). Unlike these neighbors, the growth tolerance temperature range of strain TRM70308^T^ is 15–45 °C, with a pH range of 6–10 and NaCl tolerance of 0–10%. The optimal growth conditions for strain TRM70308^T^ are 37 °C, 1% NaCl, and pH 7. The strain can utilize several sugars, including lactose, L-arabinose, sucrose, D-mannitol, glucose, D-fructose, and L-rhamnose, but cannot use raffinose, xylose, or meso-inositol as the sole carbon source. Positive results were obtained for urease, nitrate reduction, cellulose hydrolase, and starch hydrolase activity. Conversely, the results of gelatin liquefaction and catalase production tests were negative (Table S1).

The results of the comparative analysis of the whole-cell hydrolyzed sugar composition, whole-cell hydrolyzed amino acid composition, and polar lipid fractions of strain TRM70308^T^ and similar strains are presented in Table 1. The hydrolyzed sugars of strain TRM70308^T^ were consistent with those found in *S. chumphonensis* KK1-2^T^, *S. lycii* TRM66187^T^, and *S. durbertensis* NEAU-S1GS20^T^, which were classified as ribose and xylose. The amino acid types identified were _LL_-DAP and _Meso_-DAP, whereas the polar lipids included phosphatidylethanolamine (PE), phosphatidylcholine (PC), phosphatidylinositol (PI), phosphatidylglycerol (PG), and two unidentified lipids (Figure S7). The predominant quinone types identified were MK-9 (H_6_), MK-9 (H_8_), and MK-10 (H_2_), similar to those observed in *S. durbertensis* NEAU-S1GS20^T^ (Figure S8). The predominant fatty acids in strain TRM70308^T^ were anteiso-C_15:0_ (33.87%), anteiso-C_17:0_ (28.77%), and anteiso-C_17:1_ (19.46%) (Table 1).

*Streptomyces xanthus* (xan’thus. N.L. masc. adj. *xanthus* (Gr. masc. adj. *xanthos*), yellow), Strain TRM70308^T^ was deposited at the China Center for Type Culture Collection (CCTCC) and the public BCCM/LMG Bacteria Collection under accession numbers CCTCC AA 2023002 and LMG 33103, respectively.

### 3.4. Novel Desert Strain Harbor Diverse and Unique BGCs

**Figure 4:**
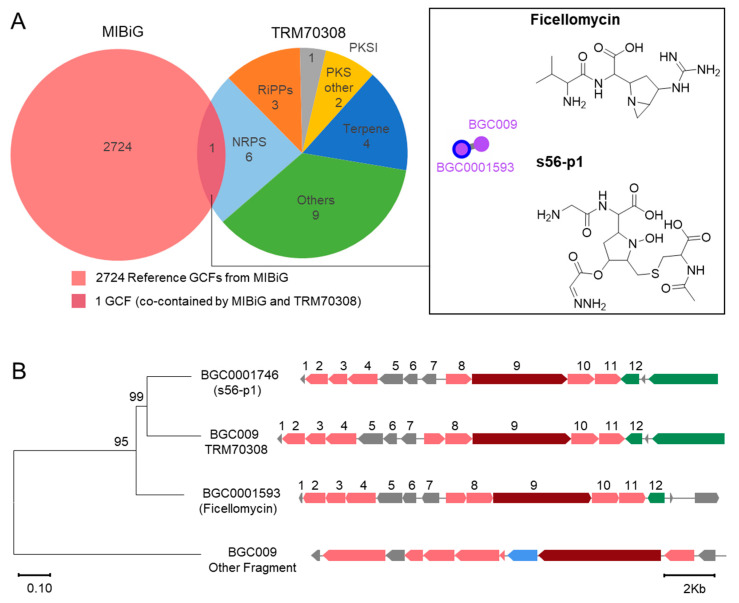
BGCs detected in TRM70308^T^. (**A**) The Venn diagram shows the overlap of experimentally validated biosynthetic gene clusters (BGCs) in the MIBiG database with those from TRM70308^T^. Only BGC009 from TRM70308^T^ overlaps with a reference BGC (Ficellomycin), forming a gene cluster family (GCF), s56-p1 represent the gene cluster corresponding to BGC009 in antiSMASH. (**B**) An evolutionary analysis of BGCs that overlap with TRM70308^T^ BGC009, based on data from the MIBiG database and antiSMASH prediction results, bootstrap values (expressed as percentages of 1000 replications) of above 50% are shown at branch points.

Strain TRM70308^T^ was predicted by antiSMASH to contain 25 BGCs encoding eight types of natural products: RiPPs (*n* = 3), terpenoids (*n* = 4), NRPS (*n* = 6), type I PKS (*n* = 1), other PKS (*n* = 2), and others (*n* = 9). Among these, there are PKS-NRPS hybrids (*n* = 2) and other hybrids (*n* = 8). According to BiG-SCAPE grouping, all 25 BGCs were distinct gene cluster families (GCFs), with only one NRPS type (GCF009) categorized as a GCF with reference BGCs in the MIBiG database (Figure 4A). The remaining BGCs exhibited unique structures and lower similarity modules (5–20%), highlighting the distinctiveness of most BGCs within the TRM70308^T^ genome (Figure S9). Further examination of GCF009, associated with BGC0001593 in the MIBiG database, revealed its association with Ficellomycin. However, the gene cluster corresponding to GCF009 in antiSMASH is BGC0001746, with the predicted product being s56-p1. For evolutionary analysis, 12 genes with the same function in the three BGCs, including BGC0001593 and BGC0001746, were linked in tandem. The remaining genes in BGC009 were used as exogenous sequences. The results indicated that BGC009 is closer to BGC0001746 (Figure 4B). The biosynthetic pathways initiated by s56-1 and Ficellomycin reportedly originate from the same amino acid unit, with Ficellomycin forming s56-1 through the action of amide P450 enzymes [35].

### 3.5. Metabolomics Confirmed BGC Expression and Detected Unannotated NPs

**Figure 5:**
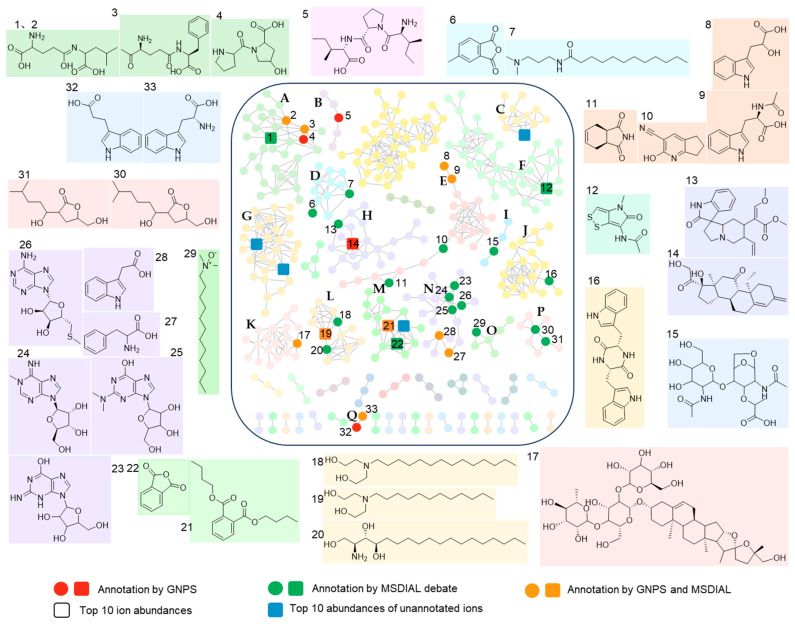
FBMN analysis of TRM70308^T^ Metabolomic after 5 days of culturing on ISP4. Each node in the molecular network represents a precursor ion, with A–Q indicating networks containing annotated nodes. Different networks are depicted in various colors, representing different types of metabolites. Corresponding compound structures are highlighted with matching background colors.

To explore the diversity of secondary metabolites, an untargeted mass spectrometry-based metabolomics approach was used to assess the metabolome of TRM70308 ^T^ in crude ISP3 medium extract samples. Through tandem mass spectrometry (MS/MS) spectral denoising and comparison with the Global Natural Products Social Molecular Networking (GNPS) online platform, 437 features were identified, resulting in 90 clusters. Among these, 36 networks contained fewer than 10 nodes, and 41 single nodes were removed from the network graph, suggesting the detection of many structurally distinct compounds (Figure 5). Following extensive database searches, manual inspection, and other chemoinformatics predictions, 33 nodes were annotated into 13 categories (Figure S9). Of these, four compounds were annotated by MS/MS features in the feature-based molecular network (FBMN) (Figure S10), 28 compounds were annotated by other databases according MSDIAL (Figures S11–S29), and 10 compounds were co-annotated by the FBMN and other databases (Figures S30–S39). The majority of the molecular features did not match known compounds. The annotated compounds included Thiolutin, an organonitrogen compound with broad-spectrum antimicrobial activity, two saponins with antifungal activity, and a range of peptides, indoles, and fatty acids (Figure 5).

The top 10 compounds detected in terms of ion abundance included peptides, alkaloids, saponins, benzoic acids, benzofurans, and four unknown types of compounds distributed across three networks. We identified the BGC expressing Thiolutin in the genome of TRM70308^T^, an NRPS-type alkaloid. Additionally, we identified seven key enzymes of the mangiferolic acid pathway that may be involved in the synthesis of benzoates and benzofurans (Table S2). Our analysis did not reveal any saponin-related genes or gene clusters. The two annotated saponins appear to have originated from the oat media rather than the strain. The detected peptides and indoles may have originated from the tryptophan metabolic pathway [36].

### 3.6. Activity Assay of Crude Extract of Fermentation Broth

The crude extract of strain TRM70308^T^ was evaluated for preliminary antimicrobial activity against fungi (*Candida albicans* ATCC 64550), gram-negative bacteria (*Escherichia coli* ATCC 25922), and gram-positive bacteria (*Staphylococcus aureus* ATCC 6538) using the filter paper agar diffusion method. The results indicated that the methanol extract exhibited activity against all three pathogens, demonstrating the most significant inhibition against *C. albicans* ATCC 64550 (26.1 ± 0.36 mm), followed by *E. coli* (24.0 ± 0.17 mm), and *S. aureus* (9.42 ± 0.40 mm) (Table S3), while the aqueous extract showed no activity against any of the tested pathogens (Figure S40). Methanol served as the control and exhibited no antimicrobial activity.

### 3.7. Isolation and Structure Identification

**Figure 6:**
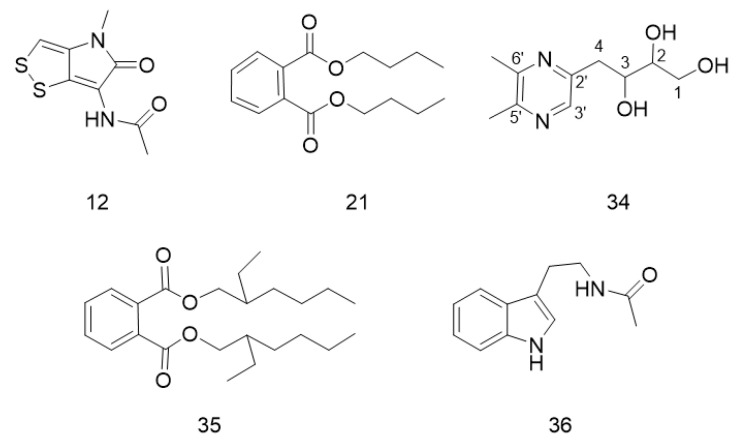
Structures of compounds isolated from TRM70308^T^.

The crude extract of strain TRM70308^T^ was enriched using D101 macroporous resin, followed by elution with methanol and further enrichment using Sephadex LH-20 and HPLC column chromatography, yielding compounds **12**, **21**, **34**, **35**, and **36**. The structures of these five compounds are illustrated in Figure 6.

Compound **12**: yellow solid. ^1^H NMR (500 MHz, DMSO-d6): δ 9.98 (1H, s), 7.34 (1H, s), 3.26 (3H, s), 2.03 (3H, s). ^13^C NMR (125 MHz, DMSO-d6): δ 24.2, 29.4, 112.7, 116.7, 134.2, 137.8, 168.0, 170.7. HRESIMS *m*/*z* 229.0096 [M + H] ^+^ (calcd for C_8_H_8_N_2_O_2_S_2_^+^, 229.0195). The ^1^H-NMR, ^13^C-NMR, HSQC, and HMBC spectra of compound **12** are shown in Figures S41–S44. In combination with the relevant literature, it was identified as Thiolutin.

Compound **21**: brown-red oil. ^1^H NMR (500 MHz, CD3OD): δ 7.71 (2H, dd), 7.61 (2H, dd), 4.29 (4H, t), 1.70 (4H, m), 1.44 (4H, m), 0.97 (6H, t). ^13^C NMR (125 MHz, CD3OD): δ 169.32, 133.60, 132.38, 129.87, 66.66, 31.73, 20.26, 14.42. HRESIMS *m*/*z* 279.1591 [M + H] ^+^ (calcd for C_16_H_22_O_4_^+^, 279.1596) further corroborates the molecular identification. The ^1^H-NMR and ^13^C-NMR spectra of compound **21** are presented in Figures S45 and S46. This compound was identified as Dibutyl phthalate(DBP) through a combination of spectroscopic data and previous literature reports [37].

Compound **34**: white powdery solid. The ^1^H NMR (500 MHz, CD3OD) data showed the presence of one olefinic methyl pyrimidine (δ 8.22 (s, 1H)), two methyl groups (δ 2.51 (s, 3H) and 2.54 (s, 3H)), and a pair of methylene groups (δ 3.12 (dd, 1H) and 3.76 (dd, 1H)), four consecutive oxygenated carbon proton signals (δ 3.94 (m, 1H), 3.64 (m, 1H), 3.54 (m, 1H), and 2.84 (m, 1H)). The ^13^C NMR (125 MHz, CD3OD) data showed two sets of quaternary carbon signals at δ 153.20, 153.17, 150.96, and 141.07. The alkenyl hydrogen carbon proton at δ 8.22 in HMBC is directly correlated with the δ 141.07 carbon signals. Furthermore, protons at δ 8.22 in HSQC are remotely correlated with carbon signals at δ 152.00, 37.63, and 20.53. HRESIMS *m*/*z* 213.1235 [M + H] ^+^ (calcd for C_10_H_16_N_2_O_3_^+^, 213.1273) was detected. The 1H-NMR, 13C-NMR, HSQC, and HMBC spectra of compound **34** are shown in Figures S47–S50. Combined with the NMR data of derivatives of pyrazines with different substitutions that have been published in the literature [38], the compound was identified as 4-(5,6-dimethylpyrazin-2-yl) butane-1,2,3-triol and named Aconicarpyrazine C.

Compound **35** is a colorless oil. ^1^H NMR (500 MHz, CD3OD): δ7.71 (1H, t), 7.62 (1H, t), 4.21 (2H, m), 1.70 (1H, m), 1.43 (2H, m), 1.36 (6H, m), 1.29 (1H, m), 0.96 (6H, m). ^13^C NMR (125 MHz, CD3OD): δ 169.30, 133.61, 132.39, 129.87, 69.08, 40.17, 31.63, 30.14, 24.95, 24.04, 14.42, 11.42. The HRESIMS *m*/*z* 391.2816 [M + H] ^+^ (calcd for C_24_H_38_O_4_^+^, 391.2848) was identified. The ^1^H-NMR and ^13^C-NMR spectra of compound **35** are shown in Figures S51 and S52. In conjunction with the relevant literature, it was identified as Bis(2-ethylhexyl) phthalate (DEHP) [37].

Compound **36** is a white, powdery solid. ^1^H NMR (500 MHz, CD3OD): δ 7.55 (1H, dd), 7.34 (1H, dd), 7.11 (1H, dd), 7.09 (1H, s), 7.01 (1H, t), 3.47 (2H, t), 3.34 (2H, s), 2.96 (2H, t), 1.94 (3H, s). ^13^C NMR (125 MHz, CD3OD): δ 171.92, 136.48, 127.38, 121.77, 120.75, 118.03, 117.49, 111.65, 110.83, 40.17, 24.92, 20.94. HRESIMS *m*/*z* 202.1069 [M + H] ^+^ (calcd for C_12_H_14_N_2_O^+^, 202.1106). The ^1^H-NMR and ^13^C-NMR spectra of compound **36** are shown in Figures S53 and S54. Upon combining these spectra with relevant literature, the compound was identified as *N*-Acetyltryptamine [39].

### 3.8. MIC of Compounds and Yield of Thiolutin

The antibacterial activity of five compounds was evaluated against five pathogens causing local plant diseases. The compound exhibited inhibitory effects on the growth of all tested pathogens. The minimum inhibitory concentration (MIC) values for these pathogens are presented in Table 2. *Valsa pyri* displayed the lowest MIC value of 6.25 μg/mL, whereas *Verticillium dahlia*, *Fusarium oxysporum*, *Erwinia amylovor* and *Alternaria gaisen* demonstrated an MIC value of 12.5 μg/mL. DMSO served as the control and exhibited no antimicrobial activity.

**Figure 7:**
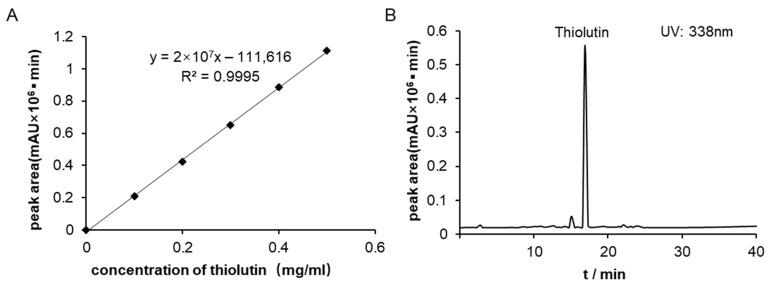
Structure and production determination of thiolutin (**A**) Standard curve of thioredoxin; (**B**) HPLC analysis of crude extract of TRM70308^T^.

The standard curve for thiolutin is represented by the equation y = (2 × 10^7^x) − 111,616, with an R^2^ value of 0.9995 (Figure 7), where x represents the peak area and y denotes the concentration of thiolutin. The R^2^ value indicates a strong linear relationship. The yields of the compounds in the crude biomass extract were analyzed with reference to a standard curve, which revealed that the yield of thiolutin was 270 mg/L.

## 4. Discussion

### 4.1. Deserts Are a Rich Source of Novel Actinomycetes

Based on genotypic, phenotypic, physiological, biochemical, and chemical characteristics, it is evident that strain TRM70308^T^ is a novel species in the genus *Streptomyces*, which is herein named *Streptomyces xanthus*. Since the first description of *Streptomyces* in 1943 [9], numerous species have been identified, making it one of the most diverse genera. Nevertheless, new *Streptomyces* species continue to be identified. Over the past decade, approximately 20 novel *Streptomyces* species have been discovered in deserts [7]. Most desert-derived *Streptomyces* exhibit anti-cancer, anti-tumor, anti-viral, anti-oxidant and anti-bacterial properties [40,41]. Desert *Streptomyces* also demonstrate a diverse array of plant growth-promoting and biocontrol properties [42,43], enhance plant resistance to environmental stress [44], and mitigate abiotic stress [42]. Desert *Streptomyces* have been used in various industrial applications. For instance, *Streptomyces fragilis* DA7-7 synthesizes a thermostable α-amylase with commercial potential in industries such as paper, food, starch saccharification, and pharmaceuticals [45]. *Streptomyces leeuwenhoekii* C34 possesses two novel oxidative enzymes with potential industrial applications [46]. This evidence supports the claim that deserts are a rich source of novel actinomycetes. This reinforces the notion that *Streptomyces* species identified in deserts can play a pivotal role in addressing challenges, such as drug resistance, scarcity of industrial raw materials, and environmental pollution.

### 4.2. Isolation and High Production of the Active Thiolutin

Compound **12**, Thiolutin, belongs to the dithiopyrrolidone class with RNAase inhibitor activity and is renowned for its broad-spectrum antibiotic efficacy against numerous gram-positive and gram-negative bacteria, including *Mycobacterium bovis*, as well as its pronounced anti-tumor activity [47]. Other members of this chemical class include Holomycin and Aureothricin. In this study, we discovered the novel biological activity of Thiolutin, which exhibited significant efficacy against several local plant pathogens, including *Verticillium dahliae*, *Fusarium oxysporum*, *Venturia pyri*, *Erwinia amylovora* (the causative agent of fire blight in pears), and *Alternaria gaisen* (responsible for black spot in pears). This is the first reported isolation of thiolutin from a desert-origin actinomycete.

Owing to significant biological properties, substantial research has been devoted to the biosynthesis of thiolutin. Although crucial biosynthetic genes have been identified [48], the synthesis of these compounds remains an active area of research [49]. A patent reported the discovery of thiolutin; however, its yield was extremely low, with only 1.8 mg isolated from 200 L of fermentation broth. Additionally, the separation medium comprises five components, necessitating complex separation procedures [50]. In this study, thiolutin was prepared from the fermentation broth of an oat culture medium in a single step, yielding 270 mg/L of thiolutin, the highest titer reported to date. The first report on the efficacy of thiolutin was published in 1958 [51], highlighting its use in spraying apple trees to control apple tree wilt caused by *E. amylovora*. In 2017, Merrouche identified thiolutin in *Saccharothrix algeriensis* and demonstrated its effectiveness in reducing *Fusarium oxysporum* [52]. Thiolutin holds significant potential for the prevention and control of plant diseases and bacterial susceptibility. In our study, the high yield and simplicity of the isolation method offer a viable economic foundation for pilot-scale production of thiolutin, facilitating its future design and development as a new generation of drugs targeting RNA polymerase.

Dithiopyrrolidones are a class of natural antibiotics with RNase inhibitory activity and are characterized by significant antibacterial, antifungal, and anticancer properties. Their unique biosynthetic and regulatory mechanisms suggest substantial potential applications, with thiolutin as a notable representative compound [48]. In 1948, Umezawa et al. isolated the first dithiopyrrolidone antibiotic, aureothricin, from *Streptomyces* sp. 26A-Chrysomycin [53]. Two years later, thiolutin was isolated from the metabolites of *Streptomyces albus* [54]. Thiolutin is a typical representative of the *N*-methyl-*N*-acylpyrrothine class of dithiopyrrolidones. Therefore, this class of compounds is also known as thiolutin analogs [48]. Subsequently, this class of compounds attracted the attention of numerous research groups. This is not only because of their unique structure and outstanding antibacterial and anti-fungal activities but also because of their unique biosynthetic and regulatory mechanisms. Many members of this family exhibit strong broad-spectrum resistance with good inhibitory activity against gram-positive and gram-negative bacteria, tumors, yeasts, fungi, and even parasites [55]. These compounds are the precursors for the design of a new generation of RNA polymerase-related drugs. Yakushiji et al. recently developed a new series of RNA polymerase inhibitors by doping holomycin into several myxopyronine backbones with good antimicrobial activity [56], implying that the use of pyrrolidines as building blocks to form heterodimeric drugs is an important direction for the development of new drugs.

### 4.3. Isolation and Activity of Other Compounds

Compounds **21** and **35** are phthalate ester (PAE) compounds. DBP and DEHP, commonly used as plasticizers and softeners in industry, are often released into the environment through industrial wastewater. This is not the first report of such compounds being isolated from microorganisms. The inhibitory effects of DBP and DEHP on pathogenic fungi and bacteria have been documented, with DBP showing greater inhibition than DEHP. DBP was among the top 10 metabolites of TRM70308^T^ in terms of ionic abundance. Studies have shown that the biosynthesis of these compounds may originate from the glucose substrate oxalic acid pathway [57]. Key enzymes of this pathway were found in tandem in the genome of TRM70308^T^, suggesting that this strain can synthesize DBP, thereby indirectly confirming that these compounds are derived from the strain rather than the medium.

Compound **34** belongs to a new class of pyrazine compounds, primarily isolated from plants, known for their hypoglycemic activity [38]. This is the first instance of such compounds being isolated from a microorganism. The biosynthetic pathway of these compounds remains unreported. Compound **36**, *N*-acetyltryptamine, has been shown to act as a partial agonist of retinal melatonin receptors. It likely originates from the tryptophan pathway, which degrades over 95% of tryptophan into various bioactive compounds [39].

The top 10 compounds by ion abundance in the metabolome analysis were grouped into six types, three of which were not annotated, while the remaining eight were grouped into five types. Compounds of the thiolutin type were isolated, and two compounds of the phthalic acid group were also isolated. The rest were not isolated, likely due to the need for more diverse separation methods. For instance, compound **1**, presumed to lack UV absorption, should be separated by TLC color development. These N-containing compounds can be detected using bismuth potassium iodide. As another example, s56-1, predicted to be acid-base unstable, requires pH adjustment to neutral during purification [58]. Furthermore, enhancing genome sequencing depth and refining the metabolic profiling database, in conjunction with transcriptome analysis, will facilitate more precise metabolomic data analysis while determining gene expression levels.

## 5. Conclusions

In this study, we identified a novel strain, *Streptomyces xanthus*, which exhibited unique genomic and metabolic profiles. Genomic and metabolomic analyses demonstrated that this strain is a source of diverse bioactive compounds. One novel compound, aconicarpyrazine C, and four known compounds were isolated from its metabolites, with all being reported for the first time from desert-derived actinomycetes. Among these, thiolutin exhibited strong activity against plant pathogenic fungi, with a production yield of 270 mg/L. These findings highlight not only the importance of desert environments in discovering microbial species and bioactive compounds, but also the potential of *S. xanthus* for pilot-scale thiolutin production. Continued exploration of desert actinomycetes may uncover additional novel metabolites and provide a strong foundation for developing innovative solutions to combat plant diseases and address challenges in agriculture and medicine.

## Figures and Tables

**Table 1:** Phenotypic and chemical characterization of strain TRM70308^T^ and its related strains.

Characteristics	1	2	3	4	5	6
Major whole-cell sugars	Glucose, Ribose	Glucose, Ribose	Glucose, Ribose	Ribose, Glucose, galactose	Glucose, Ribose, Xylose	Glucose, Ribose
Major cell-wall diamino acid	_LL_-DAP, _Meso_-DAP	_LL_-DAP	_LL_-DAP	_LL_-DAP	_LL_-DAP	_LL_-DAP
Polar lipids components	PE, PIM, GL	DPG, PI, PE, PIM, PG	DPG, PI, PE	DPG, PG, PE	DPG, PE, PI	DPG, PI, PE
Major menquinones	MK-9(H_6_), MK-9(H_8_), MK-10(H_2_)	MK9-(H_6_), MK9-(H_8_)	MK-9(H_4_), MK-9(H_6_)	MK-9(H_8_), MK-9(H_6_)	MK9-(H_6_), MK9-(H_8_)	MK–9(H_4_), MK–9(H_6_), MK–9(H_2_)
Major Fatty acid (%)						
anteiso-C15:0	33.87	19.7	16.0	-	22.1	-
anteiso-C17:0	28.77	-	-	7.7	18.1	20.1
anteiso-C17:1	19.46	-	-	4.4	-	-
iso-C15:0	-	14.2	7.1	7.2	7.2	-
iso-C16: 0	6.85	19.9	7.1	23.6	23.1	20.2
iso-C16: 1	4.01	-	-	6.3	2.6	-
iso-C17:0	-	-	3.7	3	4.9	15.1
C16:0	-	-	8.4	6.9	4.2	19.0

1. TRM70308^T^, 2. Streptomyces chumphonensis KK1-2^T^ [31], 3. Streptomyces lycii TRM66187^T^ [32], 4. Streptomyces gobiensis 1-25^T^ [33], 5. Streptomyces alkaliterrae OF1^T^ [34], 6. Streptomyces durbertensis NEAU-S1GS20^T^ [34].

**Table 2:** Bioactivity of compounds isolated from strain TRM70308^T.^

	Compounds	12	21	34	35	36	Amphotericin-B *	DMSO
Test Pathogens		MIC (µg/mL)						
*V. dahliae* ACCC 36211		12.5	-	-	-	-	3.125	-
*F. oxysporum* ACCC 31038		12.5	-	-	-	-	3.125	-
*V. pyri* WTZ-19		6.25	-	-	-	-	0.8	-
*E. amylovor* TRM-03		12.5	-	-	-	-	3.125	-
*A. gaisen* TRM-09		12.5	64	-	64	-	3.125	-

*, positive control; -, inactive at the final concentration of 100 µg/mL.

## Data Availability

GenBank accession numbers of the 16S rRNA gene sequences and draft genome of strain TRM70308^T^ are PQ368089 and JBBENY000000000, respectively.

## Supplementary Materials

The following supporting information can be downloaded at: https://www.mdpi.com/article/10.3390/biology14020164/s1, Figures S1 and S2: Phylogenetic tree of MLSA (ML, ME); Figures S3–S5: Phylogenetic tree of 16S rRNA (NJ, ML, ME); Figure S6: ANI determined using OrthoANI; Figure S7: TLC plates of polar lipids; Figure S8: HPLC analysis of menaquinones; Figure S9: Network of the BGC and types of annotated compounds.; Figure S10: Compounds identified by matching with MS/MS peaks in GNPS database; Figures S11–S29: MS/MS peak and compounds matched with MSDIAL database; Figures S30–S39: MS/MS peaks and compounds matched in both the GNPS and MSDIAL databases; Figure S40: Graph of antibacterial activity; Figures S41–S54: ^1^H NMR, ^13^C NMR, HSQC, and HMBC spectrum of compounds; Table S1: Phenotypic characterization; Table S2: Key enzymes involved in the shikimic acid pathway; Table S3: Antimicrobial activity diameter.

## References

[1] Wang T., Li F., Lu Q., Wu G., Jiang Z., Liu S., Habden X., Razumova E.A., Osterman I.A., Sergiev P.V. (2021). Diversity, novelty, antimicrobial activity, and new antibiotics of cultivable endophytic actinobacteria isolated from psammophytes collected from Taklamakan Desert. J. Pharm. Anal..

[2] Li S., Lian W.H., Han J.R., Ali M., Lin Z.L., Liu Y.H., Li L., Zhang D.Y., Jiang X.Z., Li W.J. (2023). Capturing the microbial dark matter in desert soils using culturomics-based metagenomics and high-resolution analysis. NPJ Biofilms Microbiomes.

[3] Astakala R.V., Preet G., Milne B.F., Tibyangye J., Razmilic V., Castro J.F., Asenjo J.A., Andrews B., Ebel R., Jaspars M. (2022). Mutactimycin AP, a New Mutactimycin Isolated from an Actinobacteria from the Atacama Desert. Molecules.

[4] Driche E.H., Badji B., Bijani C., Belghit S., Pont F., Mathieu F., Zitouni A. (2024). Antibacterial and antibiofilm properties of two cyclic dipeptides produced by a new desert Streptomyces sp. HG-17 strain against multidrug-resistant pathogenic bacteria. Int. Microbiol..

[5] Chen X., Li S., Zhang B., Sun H., Wang J., Zhang W., Meng W., Chen T., Dyson P., Liu G. (2022). A new bacterial tRNA enhances antibiotic production in Streptomyces by circumventing inefficient wobble base-pairing. Nucleic Acids Res..

[6] Lin L., Jing X., Lucas-Borja M.E., Shen C., Wang Y., Feng W. (2022). Rare Taxa Drive the Response of Soil Fungal Guilds to Soil Salinization in the Taklamakan Desert. Front. Microbiol..

[7] Xie F., Pathom-aree W. (2021). Actinobacteria From Desert: Diversity and Biotechnological Applications. Front. Microbiol..

[8] Mast Y., Stegmann E. (2019). Actinomycetes: The Antibiotics Producers. Antibiotics.

[9] Waksman S.A., Henrici A.T. (1943). The nomenclature and classification of the actinomycetes. J. Bacteriol..

[10] Sivalingam P., Hong K., Pote J., Prabakar K. (2019). Extreme Environment Streptomyces: Potential Sources for New Antibacterial and Anticancer Drug Leads?. Int. J. Microbiol..

[11] Rawlings B.J. (2001). Type I polyketide biosynthesis in bacteria (Part A—Erythromycin biosynthesis). Nat. Prod. Rep..

[12] Chopra I., Roberts M. (2001). Tetracycline antibiotics: Mode of action, applications, molecular biology, and epidemiology of bacterial resistance. Microbiol. Mol. Biol. Rev..

[13] Campbell W.C. (2012). History of Avermectin and Ivermectin, with Notes on the History of Other Macrocyclic Lactone Antiparasitic Agents. Curr. Pharm. Biotechnol..

[14] Horii S., Kawahara K., Kameda Y. (1972). Studies on validamycins, new antibiotics. 8. Isolation and characterization of validamycin-C, validamycin-D, validamycin-E and validamycin-F. J. Antibiot..

[15] de Voogd N.J., Cleary D.F.R., Polónia A.R.M., Gomes N.C.M. (2015). Bacterial community composition and predicted functional ecology of sponges, sediment and seawater from the thousand islands reef complex, West Java, Indonesia. FEMS Microbiol. Ecol..

[16] Yoon S.-H., Ha S.-M., Kwon S., Lim J., Kim Y., Seo H., Chun J. (2017). Introducing EzBioCloud: A taxonomically united database of 16S rRNA gene sequences and whole-genome assemblies. Int. J. Syst. Evol. Microbiol..

[17] Coil D., Jospin G., Darling A.E. (2015). A5-miseq: An updated pipeline to assemble microbial genomes from Illumina MiSeq data. Bioinformatics.

[18] Bankevich A., Nurk S., Antipov D., Gurevich A.A., Dvorkin M., Kulikov A.S., Lesin V.M., Nikolenko S.I., Son P., Prjibelski A.D. (2012). SPAdes: A New Genome Assembly Algorithm and Its Applications to Single-Cell Sequencing. J. Comput. Biol..

[19] Alanjary M., Steinke K., Ziemert N. (2019). AutoMLST: An automated web server for generating multi-locus species trees highlighting natural product potential. Nucleic Acids Res..

[20] Kumar S., Stecher G., Li M., Knyaz C., Tamura K. (2018). MEGA X: Molecular Evolutionary Genetics Analysis across Computing Platforms. Mol. Biol. Evol..

[21] Lee I., Ouk Kim Y., Park S.-C., Chun J. (2016). OrthoANI: An improved algorithm and software for calculating average nucleotide identity. Int. J. Syst. Evol. Microbiol..

[22] Meier-Kolthoff J.P., Carbasse J.S., Peinado-Olarte R.L., Göker M. (2022). TYGS and LPSN: A database tandem for fast and reliable genome-based classification and nomenclature of prokaryotes. Nucleic Acids Res..

[23] Shirling E.T., Gottlieb D. (1966). Methods for characterization of Streptomyces species. Int. J. Syst. Evol. Microbiol..

[24] Kelly D.H. (1964). Sine Waves and Flicker Fusion. Doc. Ophthalmol..

[25] Lechevalier M.P., Bievre C.D., Lechevalier H. (1977). Ecology. Chemotaxonomy of aerobic Actinomycetes: Phospholipid composition. Biochem. Syst. Ecol..

[26] Hasegawa T., Takizawa M., Tanida S. (1983). A rapid analysis for chemical grouping of aerobic actinomycetes. J. Gen. Appl. Microbiol..

[27] Minnikin D.E., Odonnell A.G., Goodfellow M., Alderson G., Athalye M., Schaal A., Parlett J.H. (1984). An Integrated Procedure for the Extraction of Bacterial Isoprenoid Quinones and Polar Lipids. J. Microbiol. Methods.

[28] Blin K., Shaw S., Augustijn H.E., Reitz Z.L., Biermann F., Alanjary M., Fetter A., Terlouw B.R., Metcalf W.W., Helfrich E.J.N. (2023). antiSMASH 7.0: New and improved predictions for detection, regulation, chemical structures and visualisation. Nucleic Acids Res..

[29] Terlouw B.R., Blin K., Navarro-Munoz J.C., Avalon N.E., Chevrette M.G., Egbert S., Lee S., Meijer D., Recchia M.J.J., Reitz Z.L. (2023). MIBiG 3.0: A community-driven effort to annotate experimentally validated biosynthetic gene clusters. Nucleic Acids Res..

[30] Shannon P., Markiel A., Ozier O., Baliga N.S., Wang J.T., Ramage D., Amin N., Schwikowski B., Ideker T. (2003). Cytoscape: A software environment for integrated models of biomolecular interaction networks. Genome Res..

[31] Phongsopitanun W., Thawai C., Suwanborirux K., Kudo T., Ohkuma M., Tanasupawat S. (2014). Streptomyces chumphonensis sp. nov., isolated from marine sediments. Int. J. Syst. Evol. Microbiol..

[32] Ma L., Zeng H., Xia Z., Luo X., Zhang L., Wan C. (2020). *Streptomyces lycii* sp. nov., an endogenous actinomycete isolated from *Lycium ruthenicum*. Int. J. Syst. Evol. Microbiol..

[33] Wen Y., Zhang G., Bahadur A., Liu Y., Zhang Z., Chen T., Liu G., Zhang W. (2022). *Streptomyces gobiensis* sp. nov., an antimicrobial producing actinobacterium isolated from soil under black Gobi rocks. Int. J. Syst. Evol. Microbiol..

[34] Świecimska M., Golińska P., Nouioui I., Wypij M., Rai M., Sangal V., Goodfellow M. (2020). *Streptomyces alkaliterrae* sp. nov., isolated from an alkaline soil, and emended descriptions of *Streptomyces alkaliphilus*, *Streptomyces calidiresistens* and *Streptomyces durbertensis*. Syst. Appl. Microbiol..

[35] He X., Li M., Song S., Wu X., Zhang J., Wu G., Yue R., Cui H., Song S., Ma C. (2018). Ficellomycin: An aziridine alkaloid antibiotic with potential therapeutic capacity. Appl. Microbiol. Biotechnol..

[36] Xue C., Li G.L., Zheng Q.X., Gu X.Y., Shi Q.M., Su Y.S., Chu Q.F., Yuan X., Bao Z.Y., Lu J. (2023). Tryptophan metabolism in health and disease. Cell Metab..

[37] Ma G., Fu H., Wu S., Bao Z., Wang S., Ge P. (2014). Isolation and structure elucidation of antifungal metabolites from marine Paenibacillus polymyxa strain L1-9. Acta Phytopathol. Sin..

[38] Guo L., Peng C., Dai O., Geng Z., Guo Y.-P., Xie X.-F., He C.-J., Li X.-H. (2013). Two new pyrazines from the parent roots of Aconitum carmichaelii. Biochem. Syst. Ecol..

[39] Pedras M.S.C., Yu Y., Liu J., Tandron-Moya Y.A. (2005). Metabolites produced by the phytopathogenic fungus Rhizoctonia solani: Isolation, chemical structure determination, syntheses and bioactivity. Z. Fur Naturforschung Sect. C-A J. Biosci..

[40] Elsayed S.S., Trusch F., Deng H., Raab A., Prokes I., Busarakam K., Asenjo J.A., Andrews B.A., van West P., Bull A.T. (2015). Chaxapeptin, a Lasso Peptide from Extremotolerant *Streptomyces leeuwenhoekii* Strain C58 from the Hyperarid Atacama Desert. J. Org. Chem..

[41] Franco Castro J., Razmilic V., Pablo Gomez-Escribano J., Andrews B., Asenjo J., Bibb M. (2018). The ‘gifted’ actinomycete *Streptomyces leeuwenhoekii*. Antonie Van Leeuwenhoek Int. J. Gen. Mol. Microbiol..

[42] Toumatia O., Compant S., Yekkour A., Goudjal Y., Sabaou N., Mathieu F., Sessitsch A., Zitouni A. (2016). Biocontrol and plant growth promoting properties of *Streptomyces mutabilis* strain IA1 isolated from a Saharan soil on wheat seedlings and visualization of its niches of colonization. S. Afr. J. Bot..

[43] Zahra T., Hamedi J., Mahdigholi K. (2020). Endophytic actinobacteria of a halophytic desert plant Pteropyrum olivieri: Promising growth enhancers of sunflower. 3 Biotech.

[44] Selim S., Hassan Y.M., Saleh A.M., Habeeb T.H., AbdElgawad H. (2019). Actinobacterium isolated from a semi-arid environment improves the drought tolerance in maize (*Zea mays* L.). Plant Physiol. Biochem..

[45] Nithya K., Muthukumar C., Kadaikunnan S., Alharbi N.S., Khaled J.M., Dhanasekaran D. (2017). Purification, characterization, and statistical optimization of a thermostable alpha-amylase from desert actinobacterium *Streptomyces fragilis* DA7-7. 3 Biotech.

[46] Gran-Scheuch A., Trajkovic M., Parra L., Fraaije M.W. (2018). Mining the Genome of *Streptomyces leeuwenhoekii*: Two New Type I Baeyer-Villiger Monooxygenases From Atacama Desert. Front. Microbiol..

[47] Jia Y., Wu S.L., Isenberg J.S., Dai S., Sipes J.M., Field L., Zeng B., Bandle R.W., Ridnour L.A., Wink D.A. (2010). Thiolutin inhibits endothelial cell adhesion by perturbing Hsp27 interactions with components of the actin and intermediate filament cytoskeleton. Cell Stress. Chaperones.

[48] Qin Z., Huang S., Yu Y., Deng H. (2013). Dithiolopyrrolone natural products: Isolation, synthesis and biosynthesis. Mar. Drugs.

[49] Chen Y., Tu Y., Pan T., Deng Z., Duan L. (2023). A Cysteine-Reloading Process Initiating the Biosynthesis of the Bicyclic Scaffold of Dithiolopyrrolones. Antibiotics.

[50] Eder C., Michael K., Joachim W. (2005). Methods of Using and Preparing Thiolutin Dioxide.

[51] Zaumeyer W.J. (1958). Antibiotics in the control of plant diseases. Annu. Rev. Microbiol..

[52] Merrouche R., Yekkour A., Lamari L., Zitouni A., Mathieu F., Sabaou N. (2017). Efficiency of Saccharothrix algeriensis NRRL B-24137 and Its Produced Antifungal Dithiolopyrrolones Compounds to Suppress Fusarium oxysporum-Induced Wilt Disease Occurring in Some Cultivated Crops. Arab. J. Sci. Eng..

[53] Umezawa H., Maeda K., Kosaka H. (2010). Isolation of a new antibiotic substance, aureothricin from a strain of strepomyces. Jpn. Med. J..

[54] Seneca H., Kane J.H., Rockenbach J. (1952). Bactericidal protozoicidal and fungicidal properties of thiolutin. Antibiot. Chemother..

[55] Li B., Wever W.J., Walsh C.T., Bowers A.A. (2014). Dithiolopyrrolones: Biosynthesis, synthesis, and activity of a unique class of disulfide-containing antibiotics. Nat. Prod. Rep..

[56] Yakushiji F., Miyamoto Y., Kunoh Y., Okamoto R., Nakaminami H., Yamazaki Y., Noguchi N., Hayashi Y. (2013). Novel Hybrid-Type Antimicrobial Agents Targeting the Switch Region of Bacterial RNA Polymerase. ACS Med. Chem. Lett..

[57] Tian C., Ni J., Chang F., Liu S., Xu N., Sun W., Xie Y., Guo Y., Ma Y., Yang Z. (2016). Bio-Source of di-n-butyl phthalate production by filamentous fungi. Sci. Rep..

[58] Matsuda K., Hasebe F., Shiwa Y., Kanesaki Y., Tomita T., Yoshikawa H., Shin-Ya K., Kuzuyama T., Nishiyama M. (2017). Genome Mining of Amino Group Carrier Protein-Mediated Machinery: Discovery and Biosynthetic Characterization of a Natural Product with Unique Hydrazone Unit. ACS Chem. Biol..

