# Supplementary material for: Metabolites Discovery from Streptomyces xanthus: Exploring the Potential of Desert Microorganisms
Source: Biology (Basel). 2025 Feb 6;14(2):164. doi: 10.3390/biology14020164 (PMC11851569; doi:10.3390/biology14020164)
Supplement: Supplementary file 1 [file biology-14-00164-s001.zip › biology-3454667-supplementary.pdf]

SUPPLEMENTARY MATERIAL

Metabolites Discovery from *Streptomyces xanthus*: Exploring the Potential of Desert Microorganisms

Xinrong Luo[1], Zhanwen Liu[1], Zhanfeng Xia[1], Xiaoxia Luo[1], Juan Zhang[2], Ailiang Chen[2], Haoxin Wang[3], Chuanxing Wan[1] and Lili Zhang[1,2]*

Table of CONTENTS

Figure S1. Phylogenetic tree of MLSA ((ML) 3
Figure S2. Phylogenetic tree of MLSA (ME).......... 4
Figure S3. Phylogenetic tree of 16S rRNA (NJ) 5
Figure S4. Phylogenetic tree of 16S rRNA (ML).......... 6
Figure S5. Phylogenetic tree of 16S rRNA (ME) 7
Figure S6. ANI determined using OrthoANI.......... 8
Figure S7. Two-dimensional TLC plates of polar lipids.. 8
Figure S8. HPLC analysis of menaquinones 8
Figure S9. Network of the BGC and types of annotated compounds.......... 9
Figure S10. Compounds identified by matching with MS/MS peaks in GNPS database 10
Figure S11-S29. Compounds identified by matching with MS/MS peaks in GNPS database.......... 10
Figure S30-S39. Compounds matched in both the GNPS and MSDIAL databases. 17
Figure S40. Graph of antibacterial activity 20
Figure S41. 1H NMR spectrum of compound 12 (500 MHz, DMSO-d6) 21
Figure S42. 1C NMR spectrum of compound 12 (125 MHz, DMSO-d6).......... 21
Figure S43. HSQC spectrum of compound 12 (500 MHz, DMSO-d6) 22
Figure S44. ^{1}HMBC spectrum of compound 12(500 MHz, DMSO-d6).......... 22
Figure S45. ^{1}H NMR spectrum of compound 21 (500 MHz, CD3OD).......... 23
Figure S46. ^{13}C NMR spectrum of compound 21 (125 MHz, CD3OD) 23
Figure S47. ^{1}H NMR spectrum of compound 34 (500 MHz, CD3OD).......... 24
Figure S48. ^{13}C NMR spectrum of compound 34 (125 MHz, CD3OD) 24
Figure S49. HSQC spectrum of compound 34 (500 MHz, CD3OD).......... 25
Figure S50. HMBC spectrum of compound 34(500 MHz, CD3OD).......... 25
Figure S51. ^{1}H NMR spectrum of compound 35 (500 MHz, CD3OD).......... 26
Figure S52. ^{13}C NMR spectrum of compound 35 (125 MHz, CD3OD) 26
Figure S53. ^{1}H NMR spectrum of compound 36(500 MHz, CD3OD).......... 27
Figure S54. ^{13}C NMR spectrum of compound 36(125 MHz, CD3OD) 27
Table S1 Phenotypic characterization of strain TRM70308^{T} and its related strains 28
Table S2. Key enzymes involved in the shikimic acid pathway in strain TRM70308 28
Table S3. Antimicrobial activity diameter.......... 28

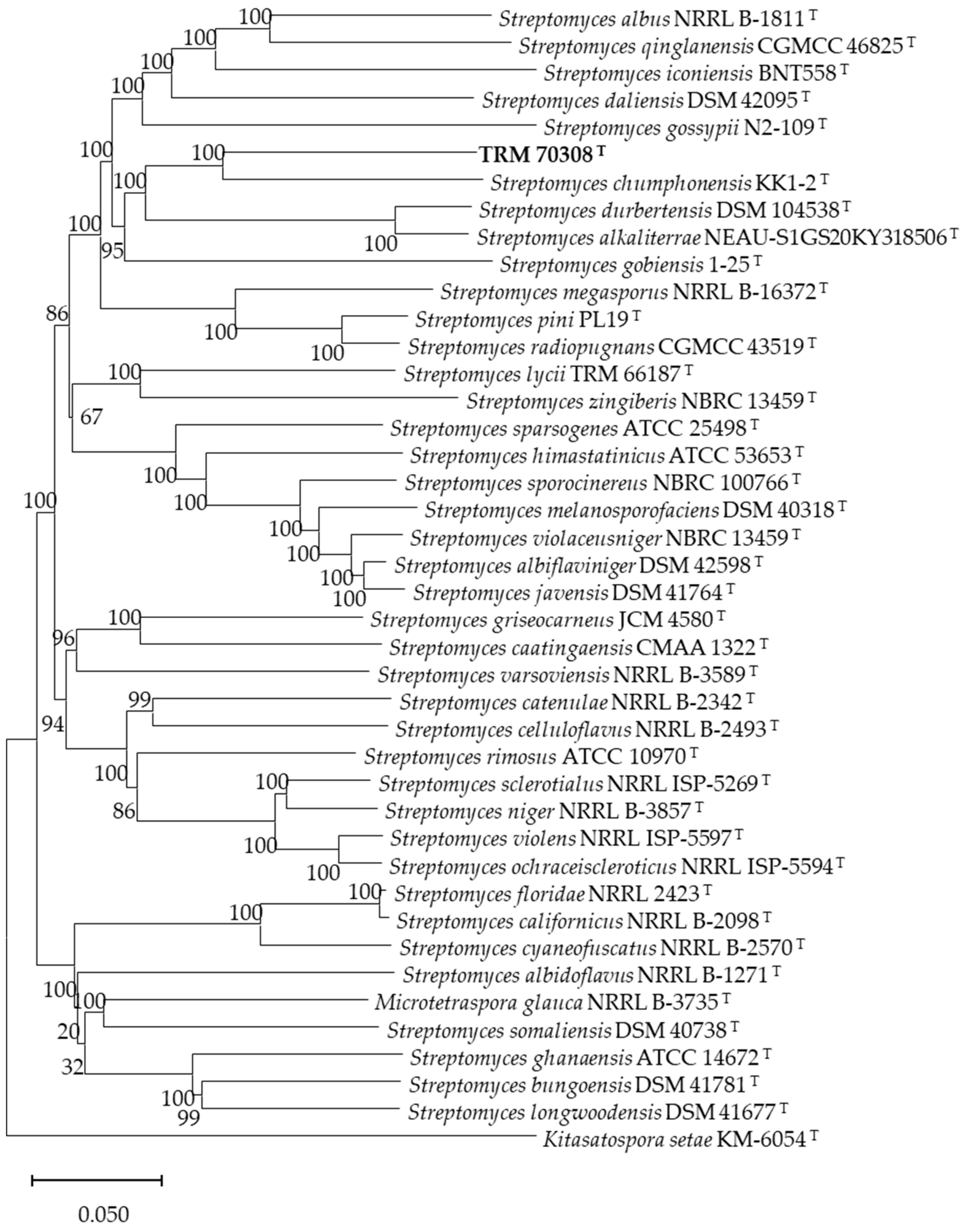

Figure S1. Phylogenetic tree of MLSA ((ML)

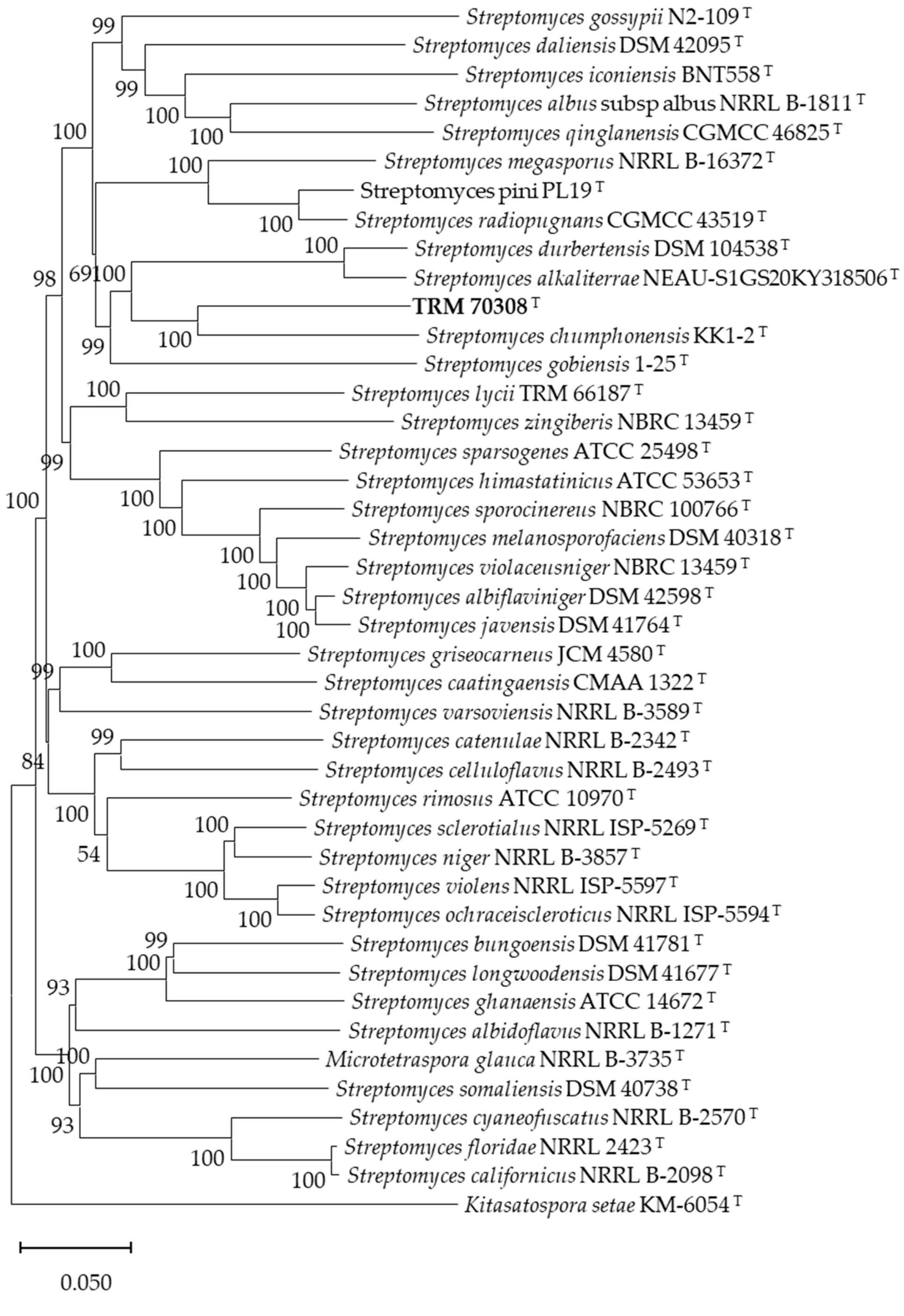

Figure S2. Phylogenetic tree of MLSA (ME)

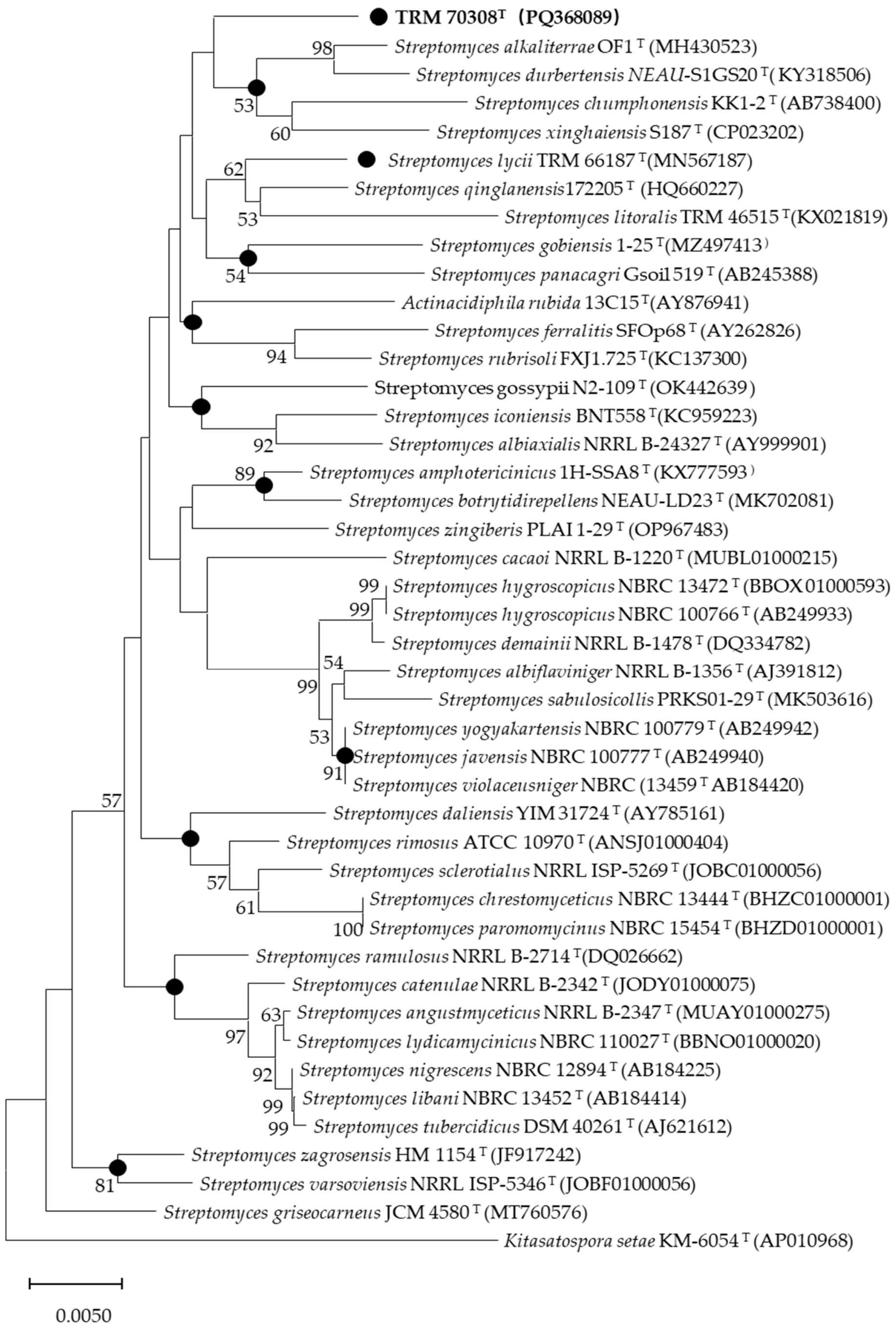

Figure S3. Phylogenetic tree of 16S rRNA (NJ)

Bootstrap percentages from 1000 replicates are indicated at the nodes, with only values greater than 50% displayed. The scale bar represents 0.005 substitutions per nucleotide position. The presence of black dots on a branch signifies that the branch also appears in Maximum Likelihood and Minimum Evolution method.

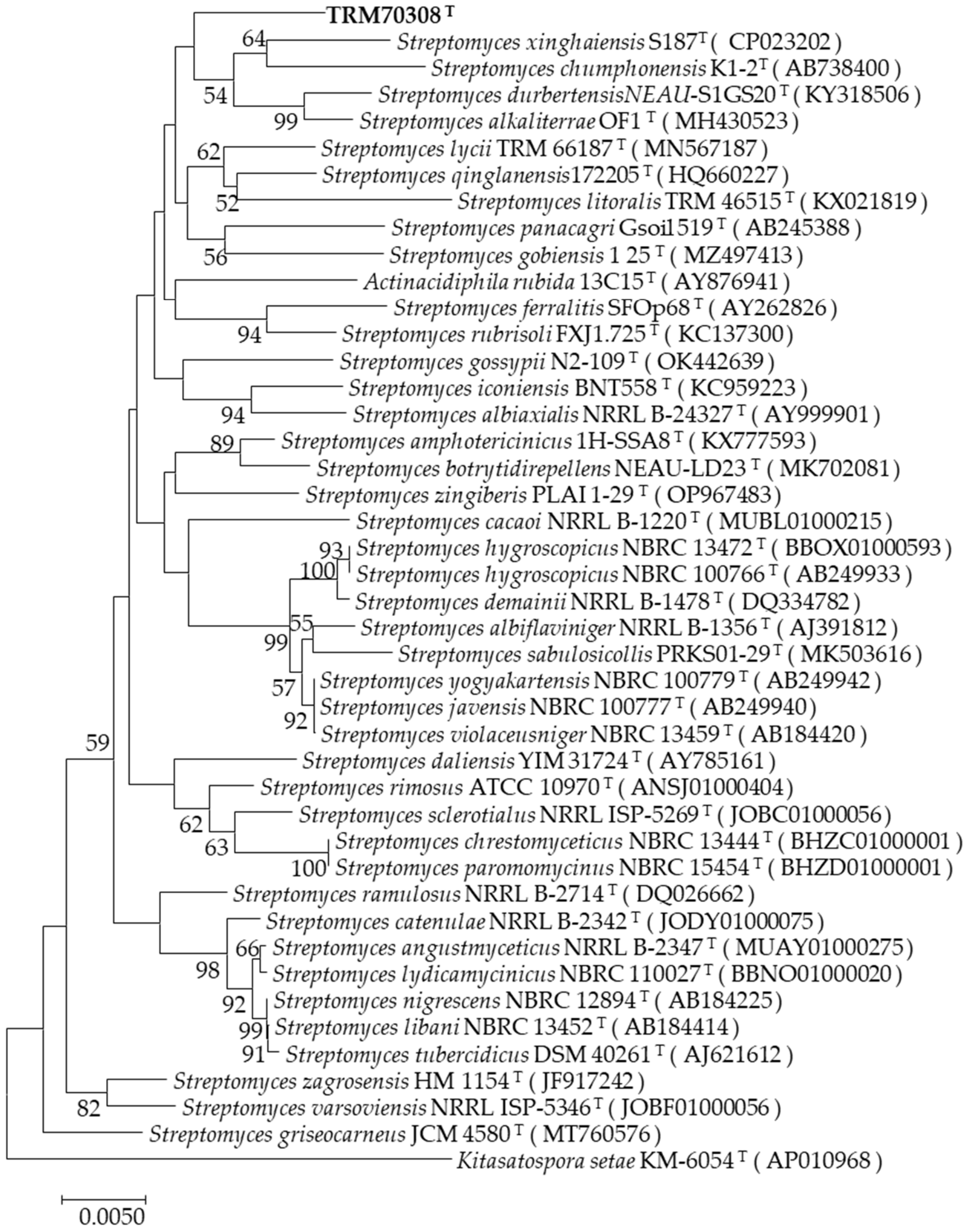

Figure S4. Phylogenetic tree of 16S rRNA (ML)

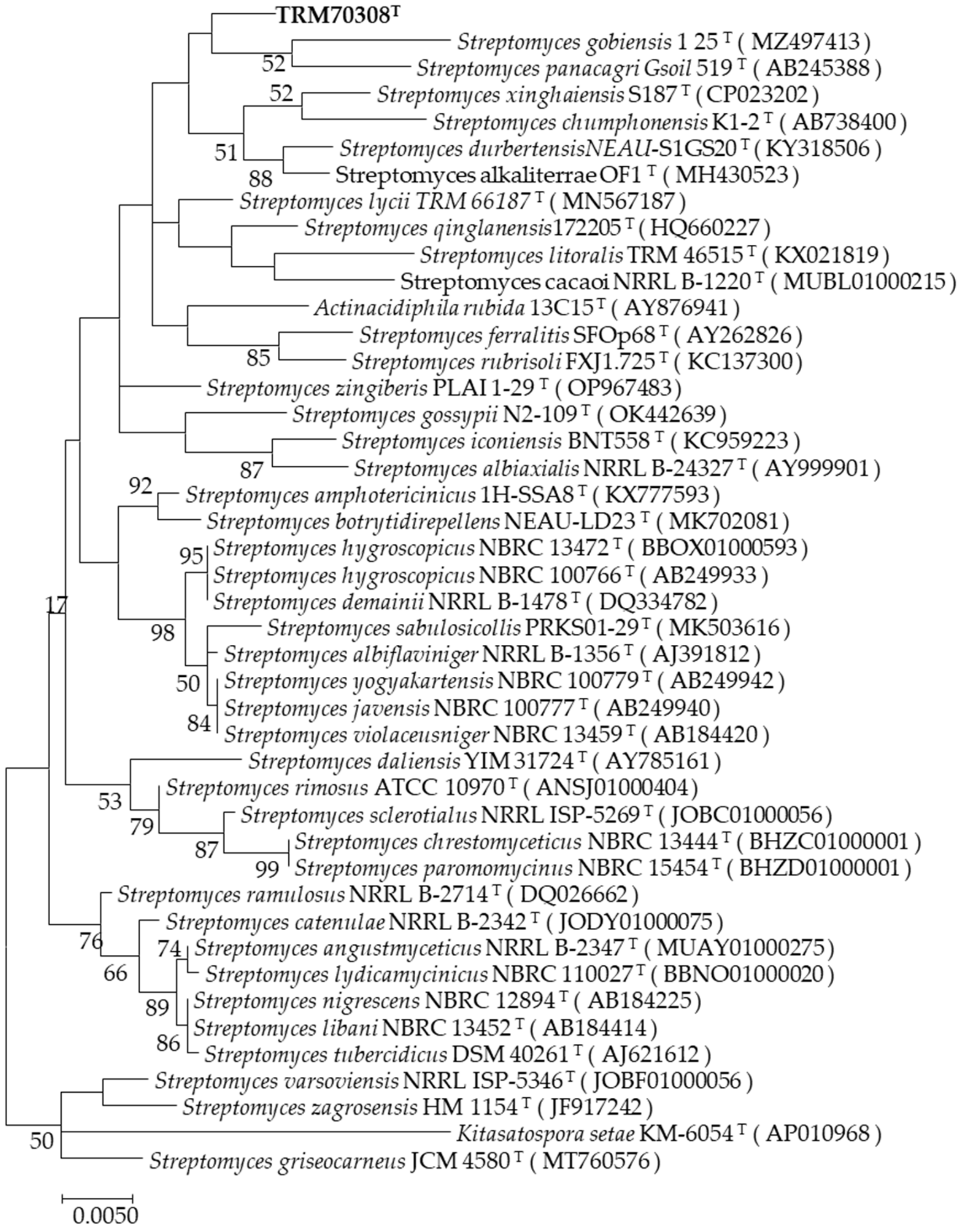

Figure S5. Phylogenetic tree of 16S rRNA (ME)

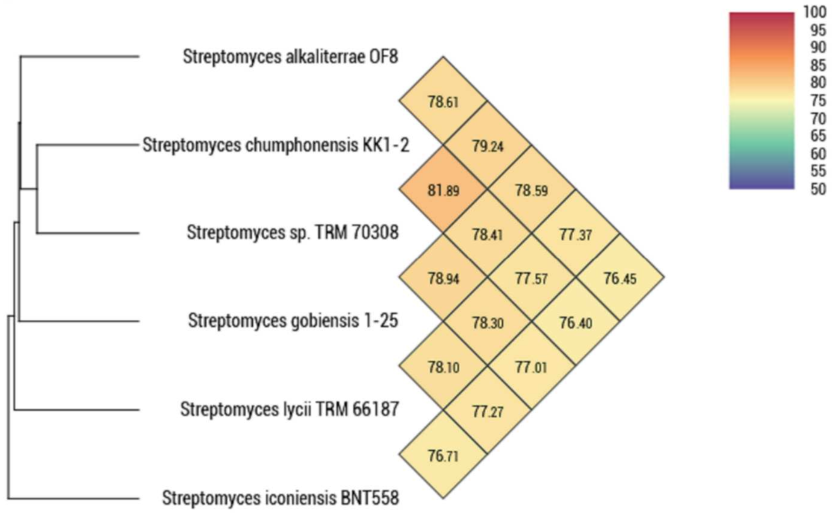

Figure S6. ANI determined using OrthoANI

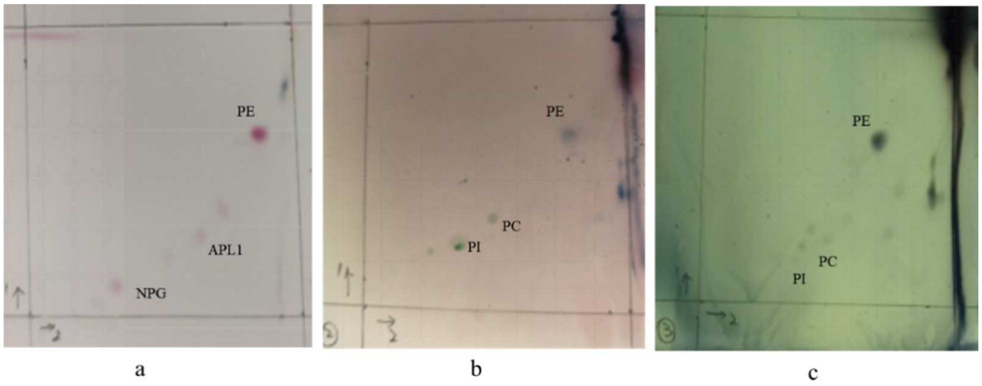

Figure S7. Two-dimensional TLC plates of polar lipids. Polar lipids after staining with molybdophosphoric acid (a), anisaldehyde (b) and ninhydrin (c).

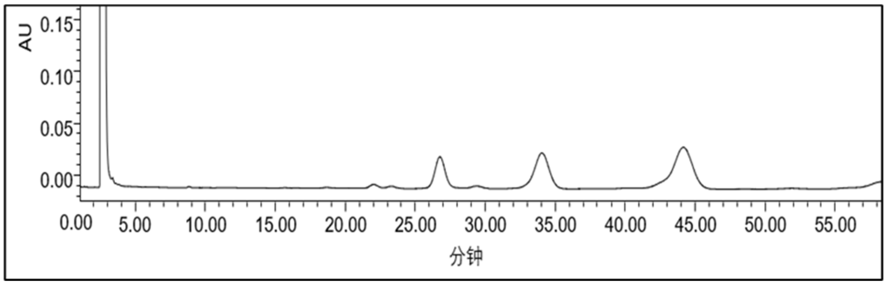

Figure S8. HPLC analysis of menaquinones

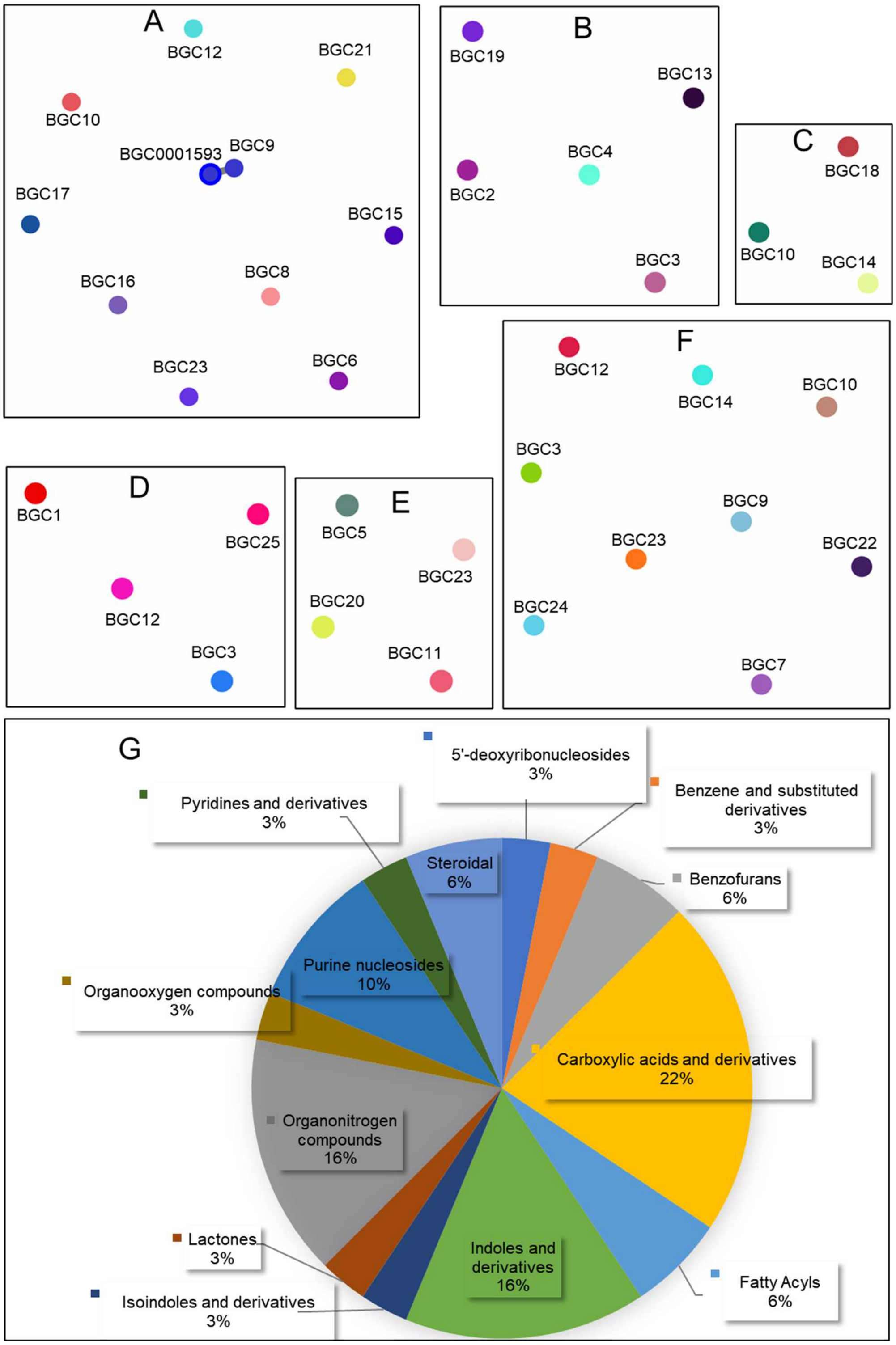

Figure S9. Network of the BGC and types of annotated compounds. A-F:The similarity networks are generated by BiG-SCAPE, their edges connect clusters that share genes (A: NRPS, B: Terpene, C: PKSI, D: PKSII, E: RiPPs, F: Others); G: 33 nodes were annotated in 13 categories of compounds.

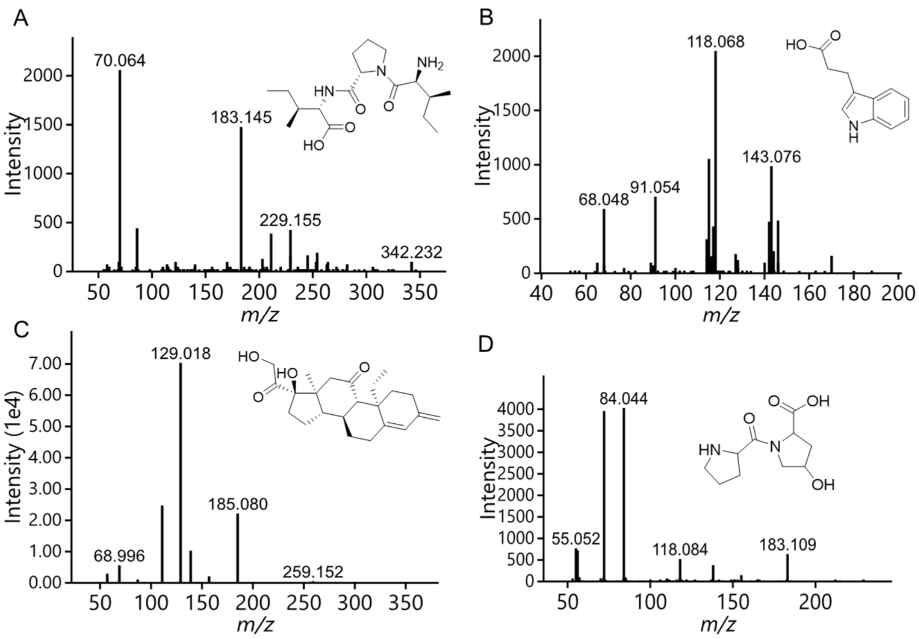

Figure S10. Compounds identified by matching with MS/MS peaks in GNPS database (A-D: compound 5, 32, 14, 4 in figure 5)

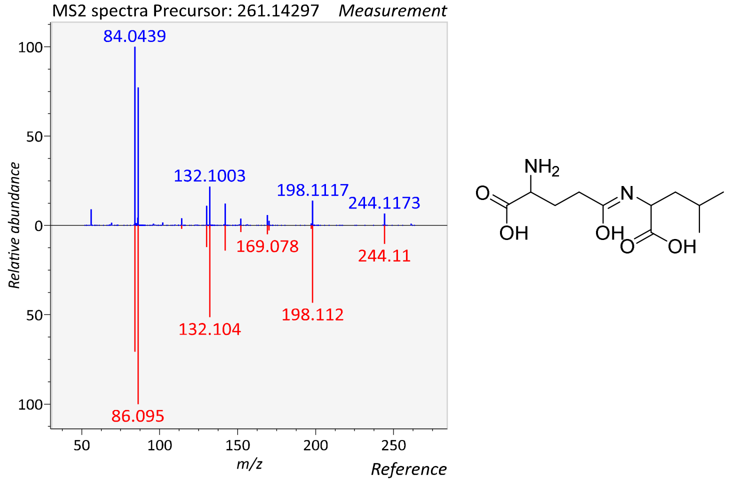

Figure S11. MS/MS peak and compound 1 matched with MSDIAL database

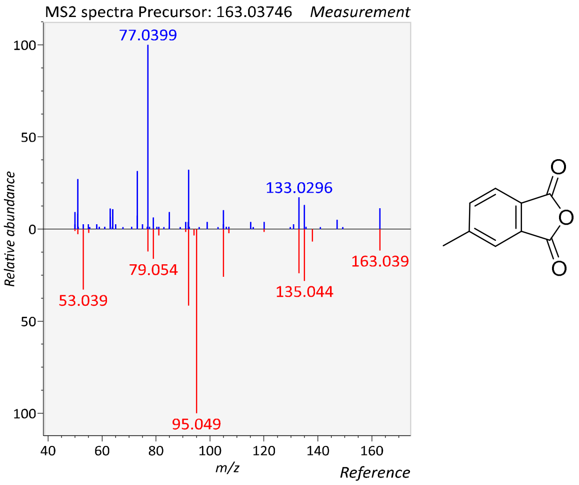

Figure S12. MS/MS peak and compound 6 matched with MSDIAL database

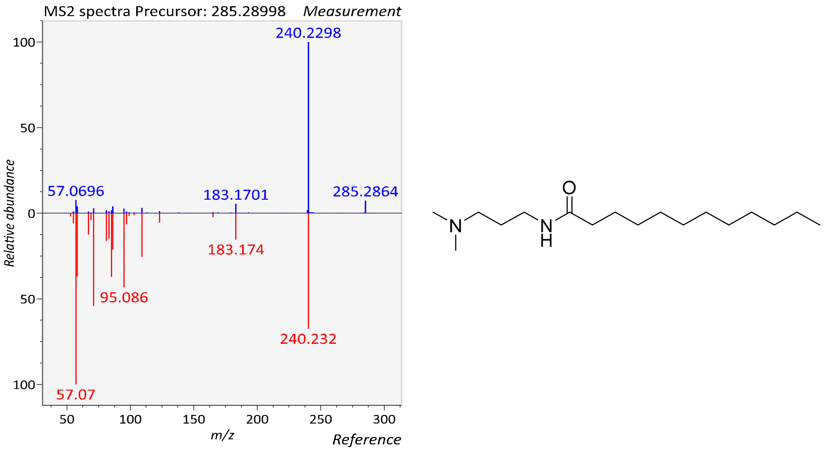

Figure S13. MS/MS peak and compound 7 matched with MSDIAL database

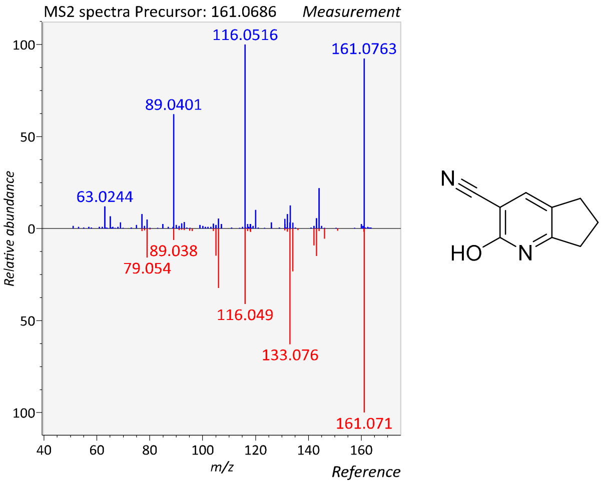

Figure S14. MS/MS peak and compound 10 matched with MSDIAL database

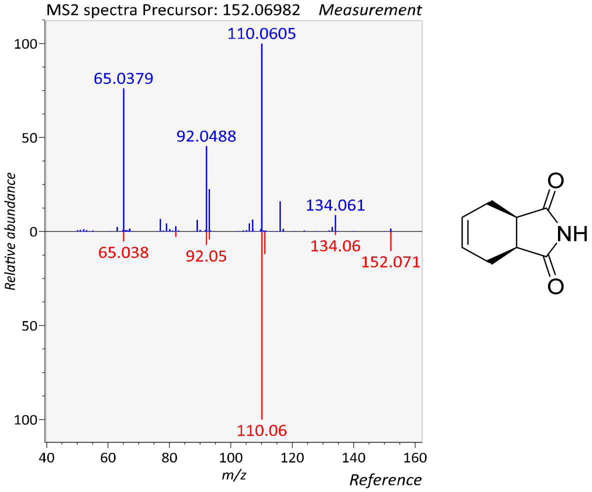

Figure S15. MS/MS peak and compound 11 matched with MSDIAL database

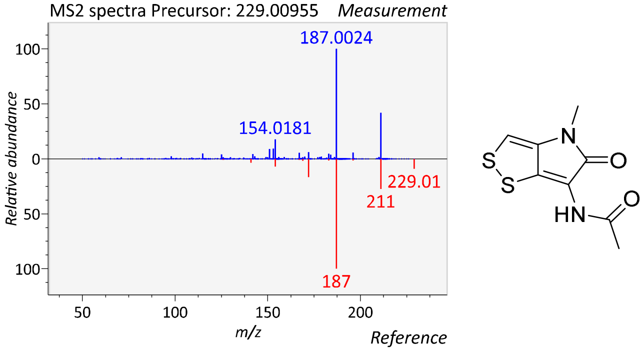

Figure S16. MS/MS peak and compound 12 matched with MSDIAL database

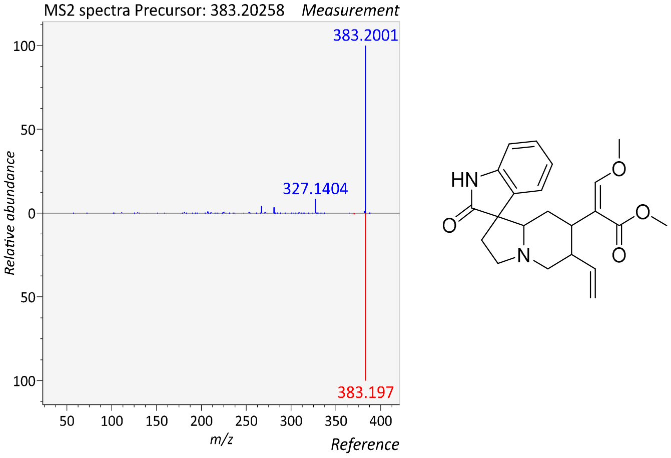

Figure S17. MS/MS peak and compound 13 matched with MSDIAL database

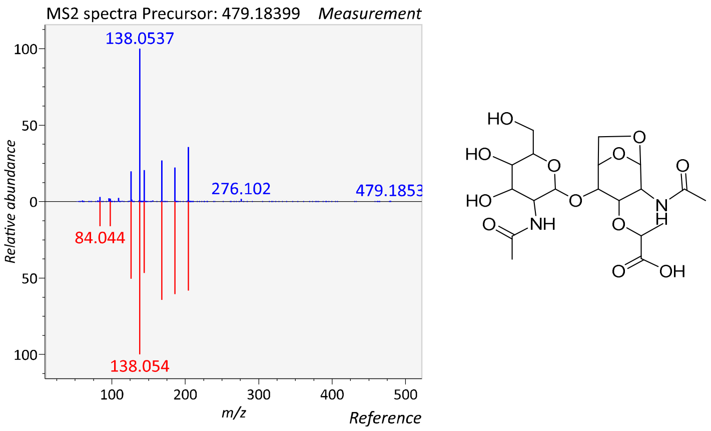

Figure S18. MS/MS peak and compound 15 matched with MSDIAL database

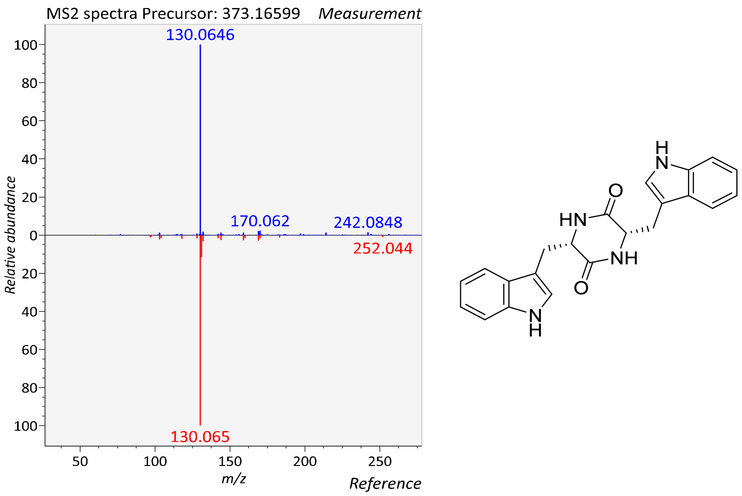

Figure S19. MS/MS peak and compound 16 matched with MSDIAL database

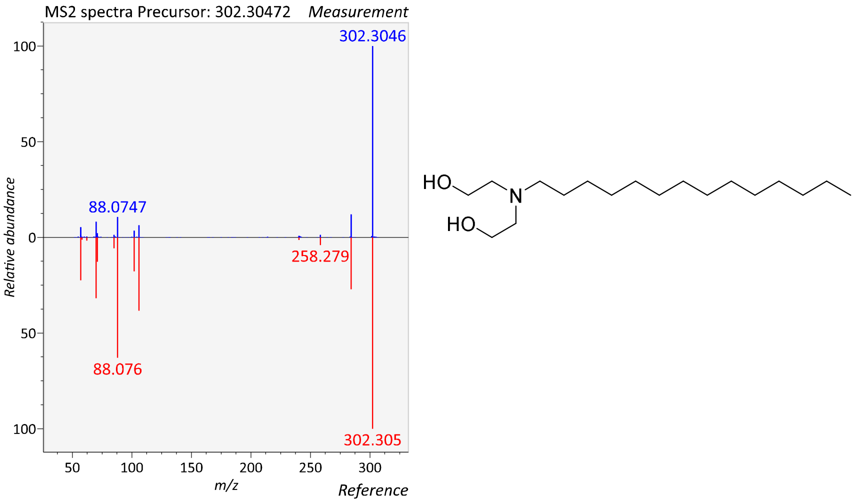

Figure S20. MS/MS peak and compound 18 matched with MSDIAL database

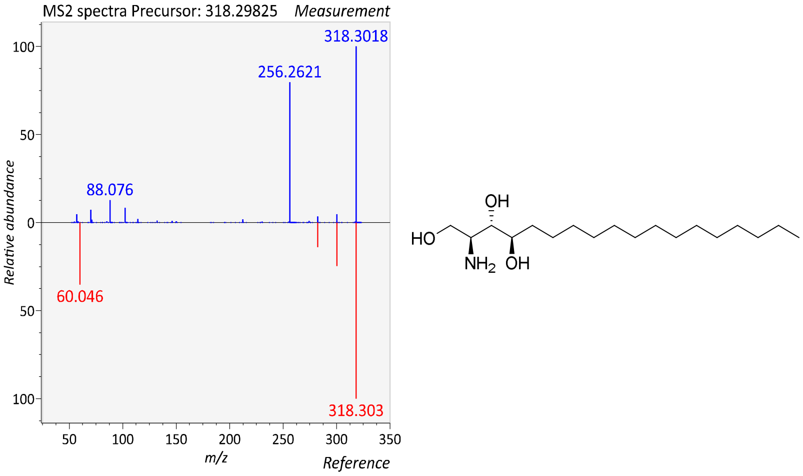

Figure S21. MS/MS peak and compound 20 matched with MSDIAL database

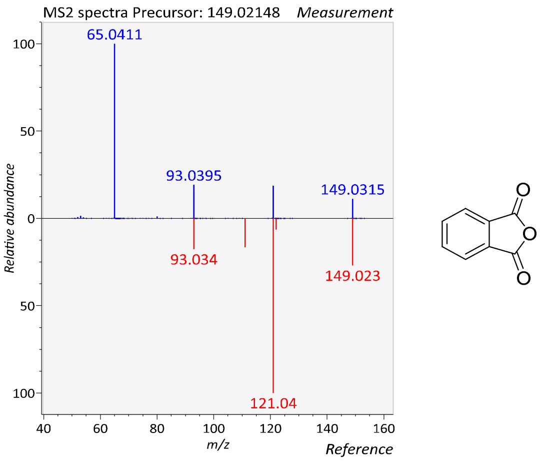

Figure S22. MS/MS peak and compound 22 matched with MSDIAL database

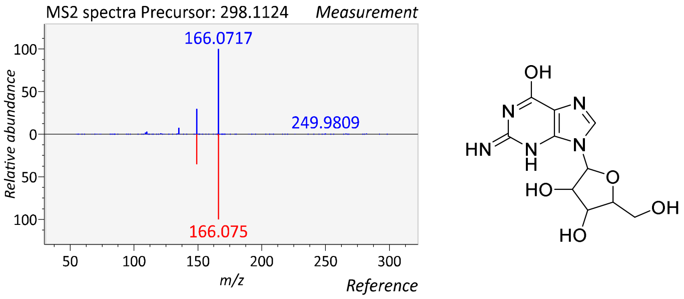

Figure S23. MS/MS peaks and compound 23 matched with MSDIAL database

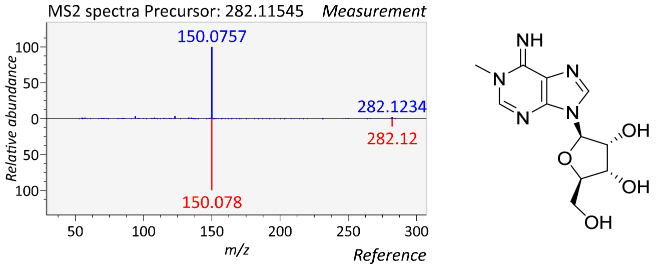

Figure S24. MS/MS peaks and compound 24 matched with MSDIAL database

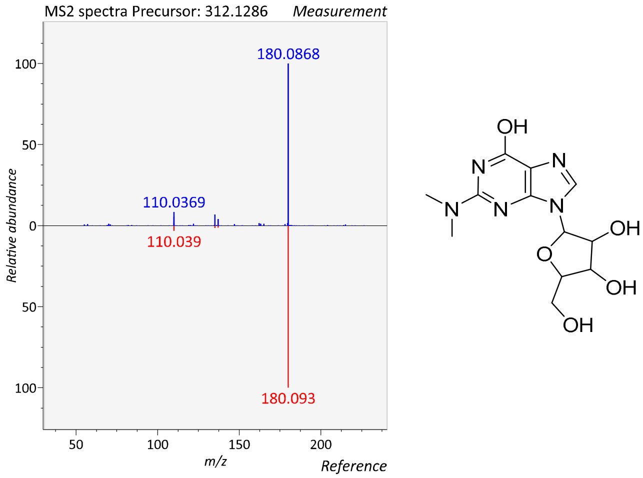

Figure S25. MS/MS peaks and compound 25 matched with MSDIAL database

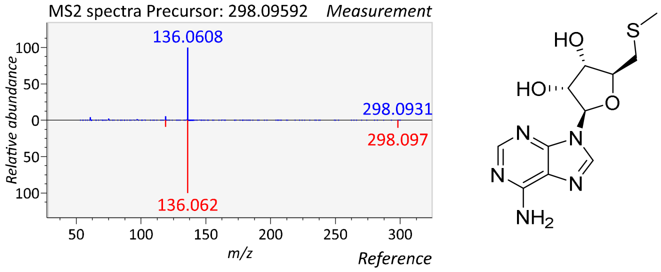

Figure S26. MS/MS peaks and compound 26 matched with MSDIAL database

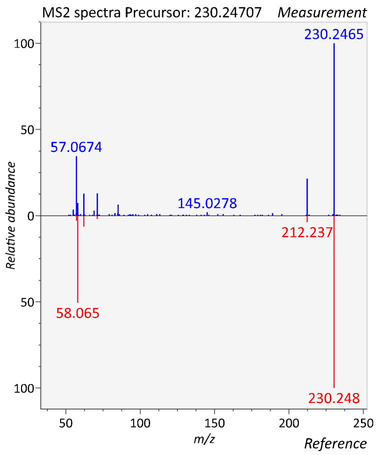

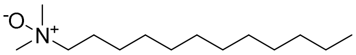

Figure S27. MS/MS peaks and compound 29 matched with MSDIAL database

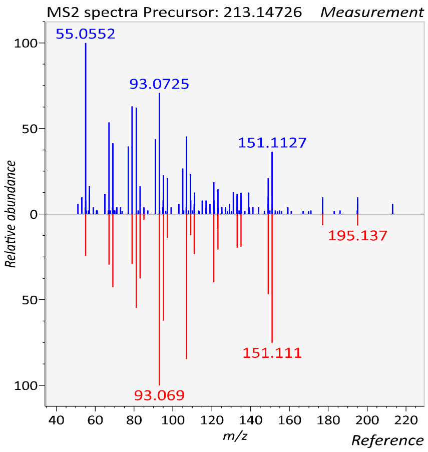

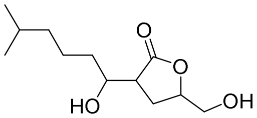

Figure S28. MS/MS peaks and compound 30 matched with MSDIAL database

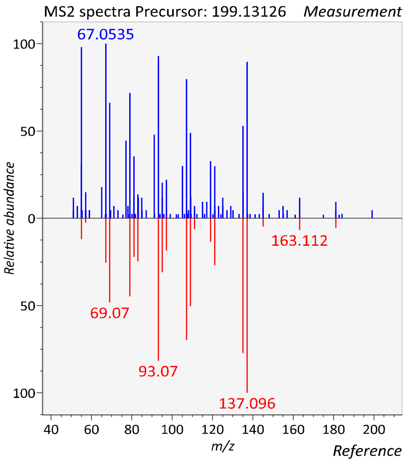

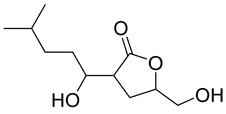

Figure S29. MS/MS peaks and compound 31 matched with MSDIAL database

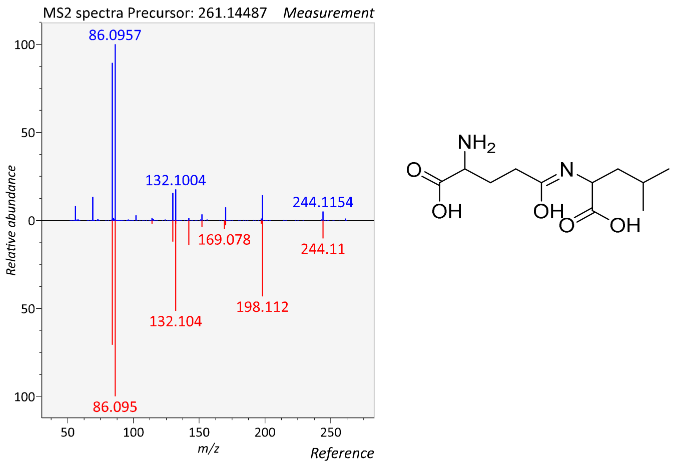

Figure S30. MS/MS peaks and compound 2 matched in both the GNPS and MSDIAL databases.

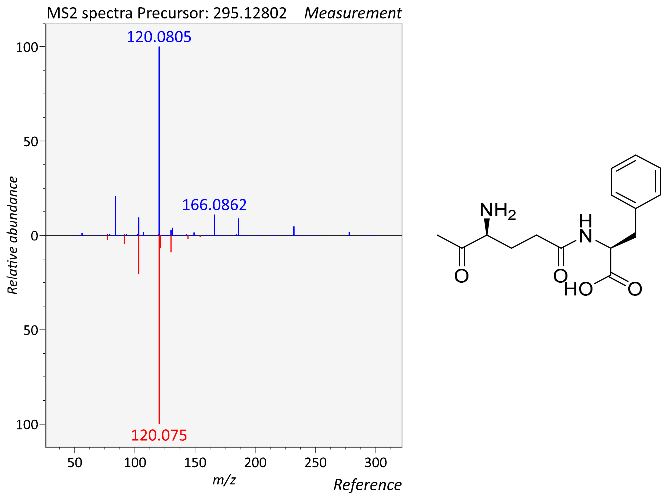

Figure S31. MS/MS peaks and compound 3 matched in both the GNPS and MSDIAL databases.

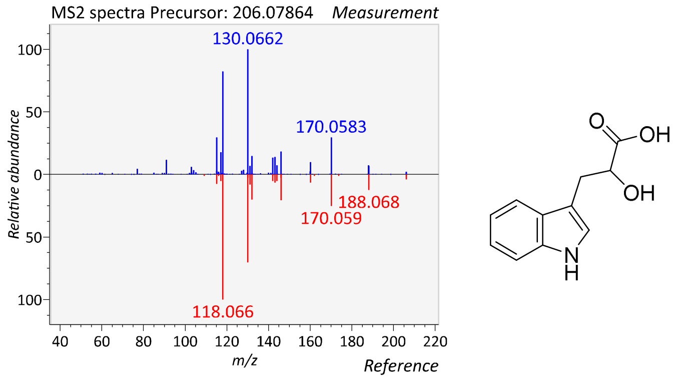

Figure S32. MS/MS peaks and compound 8 matched in both the GNPS and MSDIAL databases.

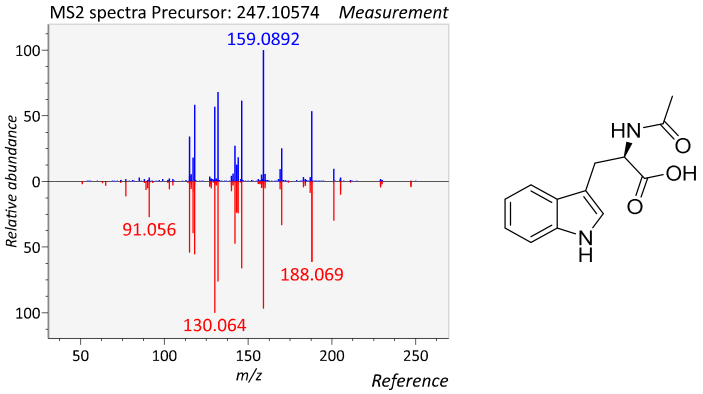

Figure S33. MS/MS peaks and compound 9 matched in both the GNPS and MSDIAL databases.

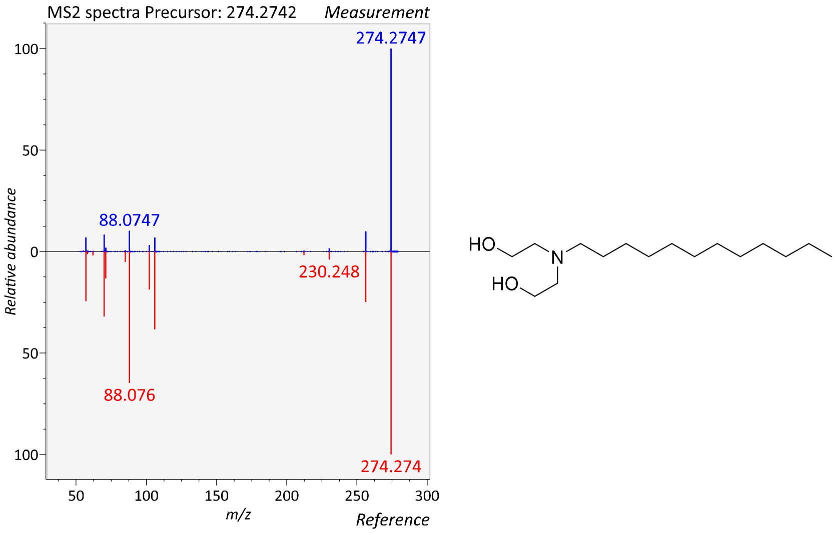

Figure S34. MS/MS peaks and compound 17 matched in both the GNPS and MSDIAL databases.

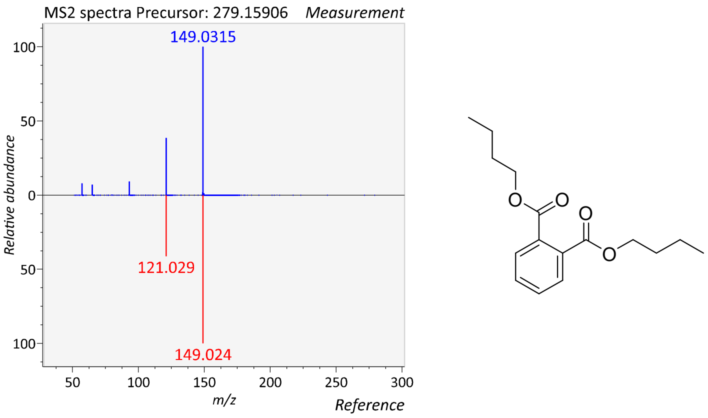

Figure S35. MS/MS peaks and compound 19 matched in both the GNPS and MSDIAL databases.

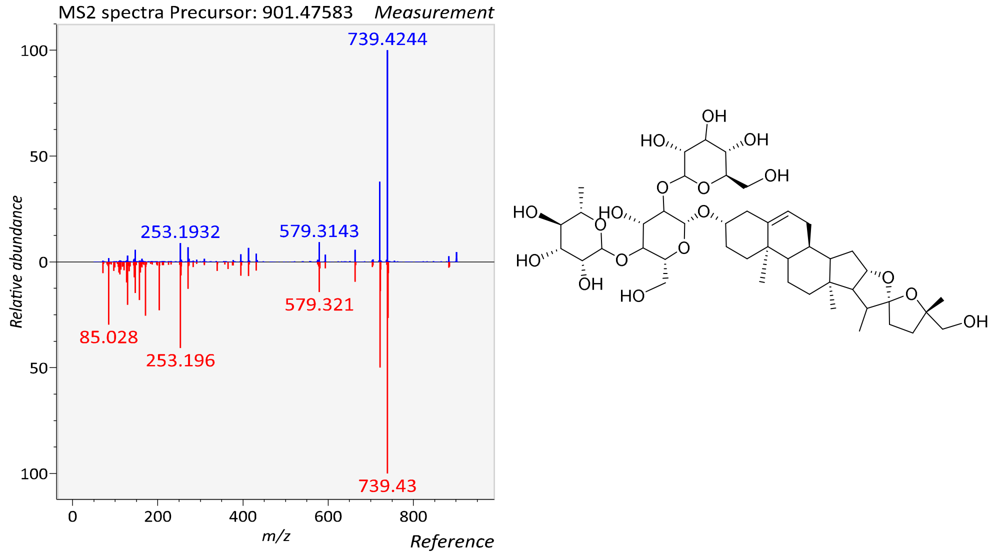

Figure S36. MS/MS peaks and compound 21 matched in both the GNPS and MSDIAL databases.

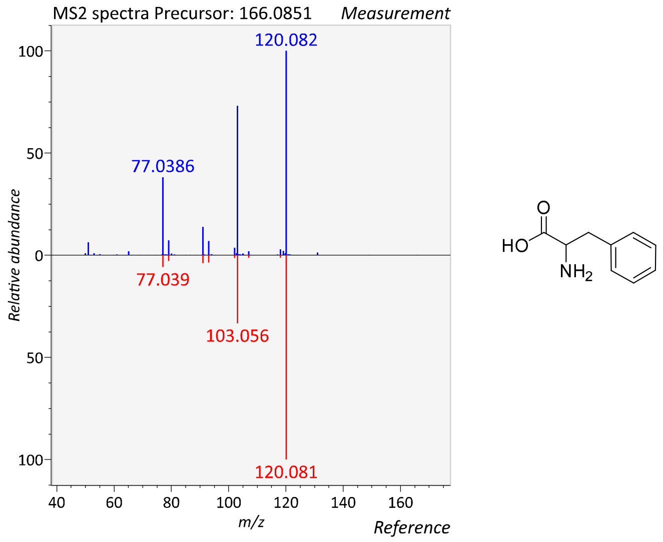

Figure S37. MS/MS peaks and compound 27 matched in both the GNPS and MSDIAL databases.

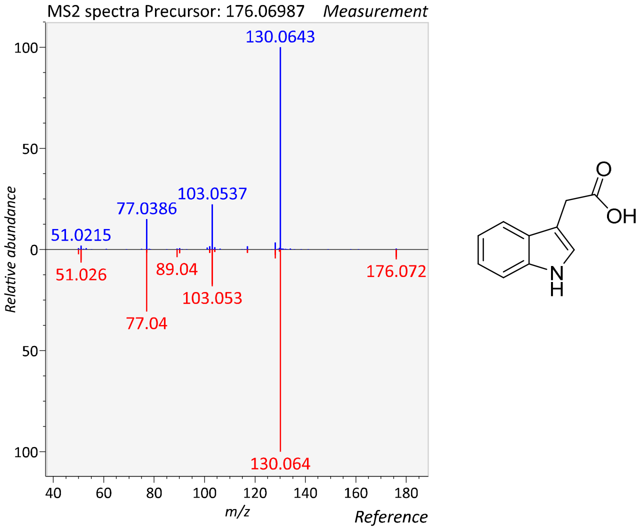

Figure S38. MS/MS peaks and compound 28 matched in both the GNPS and MSDIAL databases.

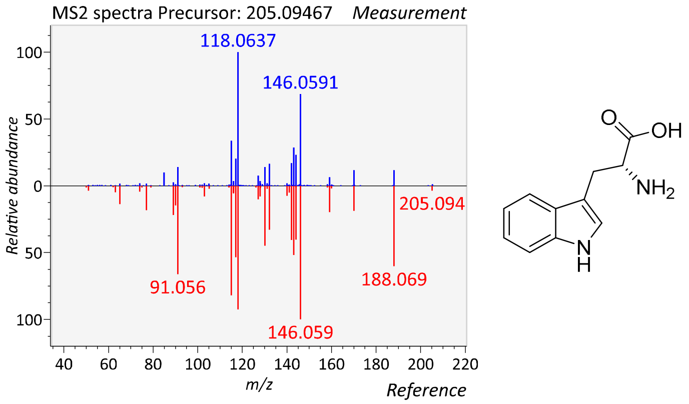

Figure S39. MS/MS peaks and compound 33 matched in both the GNPS and MSDIAL databases.

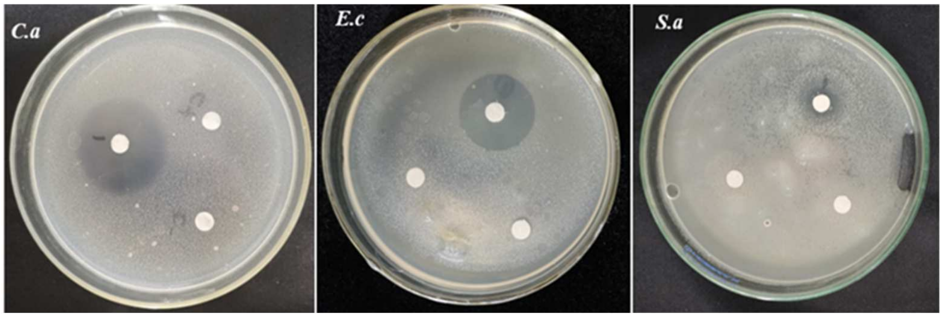

Figure S40. Graph of antibacterial activity (*C.a: Candida albicans* ATCC 64550, *E.c: Escherichia coli* ATCC 25922 and *S.a: Staphylococcus aureus* ATCC 6538)

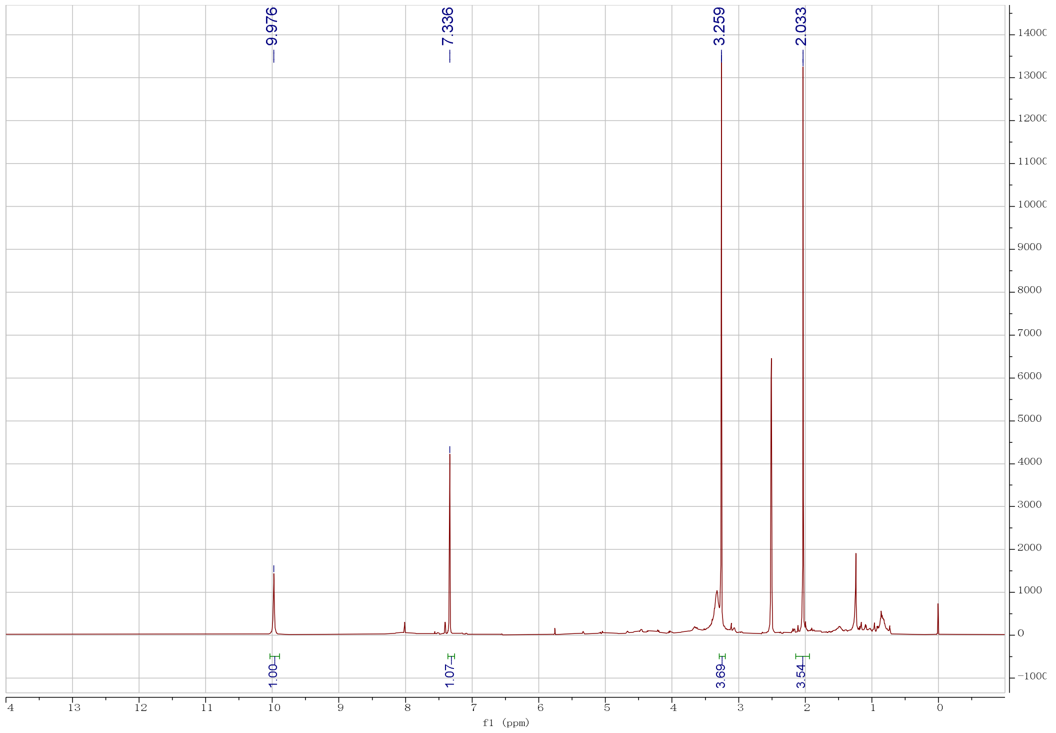

Figure S41. 1H NMR spectrum of compound 12 (500 MHz, DMSO-d6)

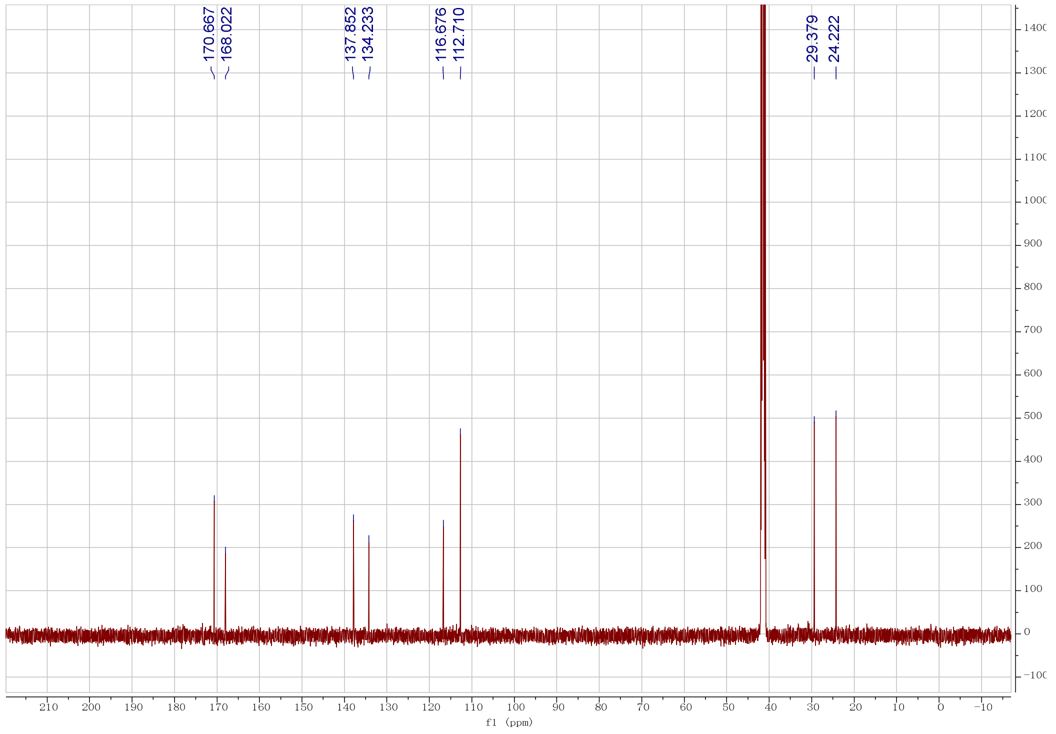

Figure S42. 1C NMR spectrum of compound 12 (125 MHz, DMSO-d6)

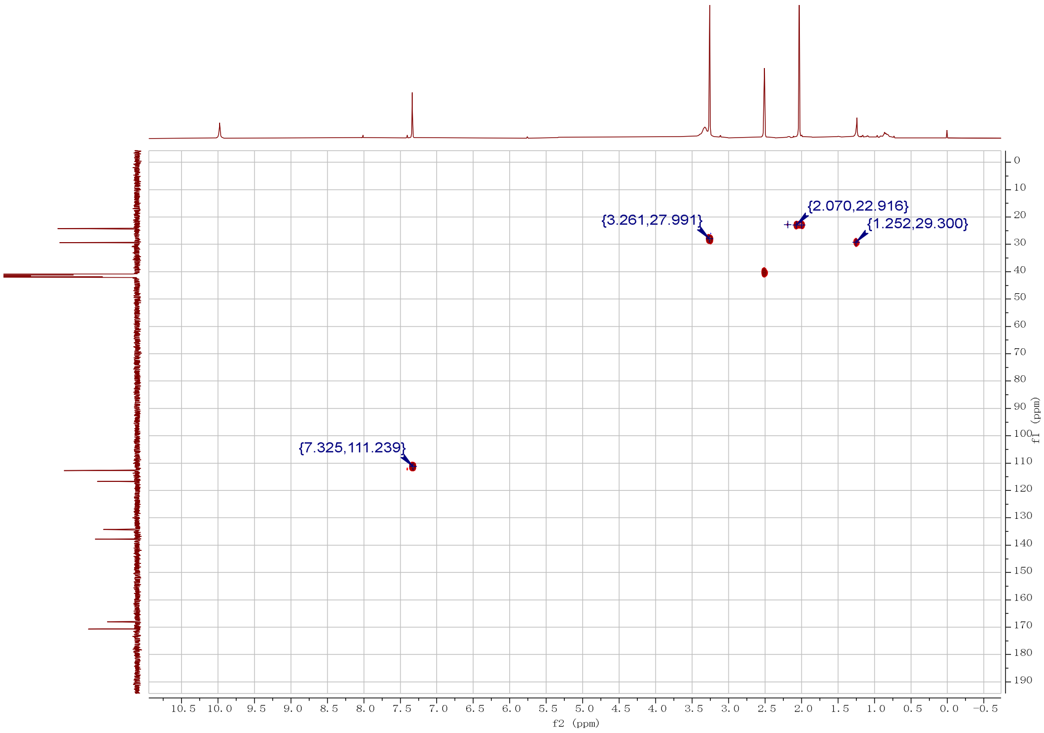

Figure S43. HSQC spectrum of compound 12 (500 MHz, DMSO-d6)

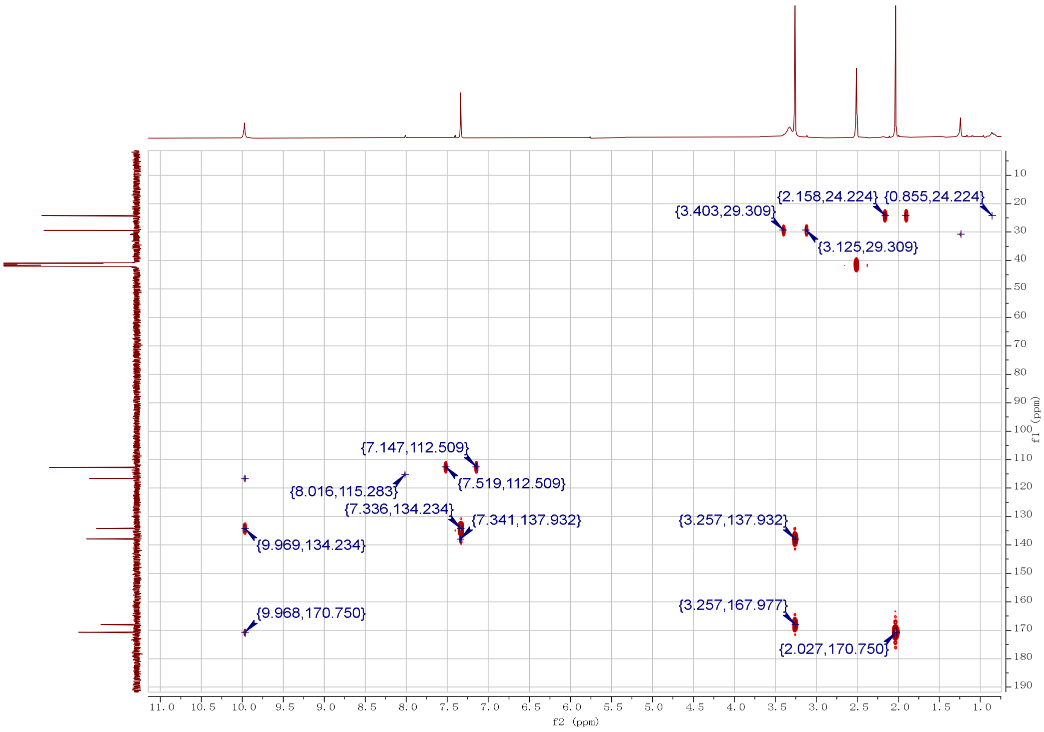

Figure S44. [1]HMBC spectrum of compound 12(500 MHz, DMSO-d6)

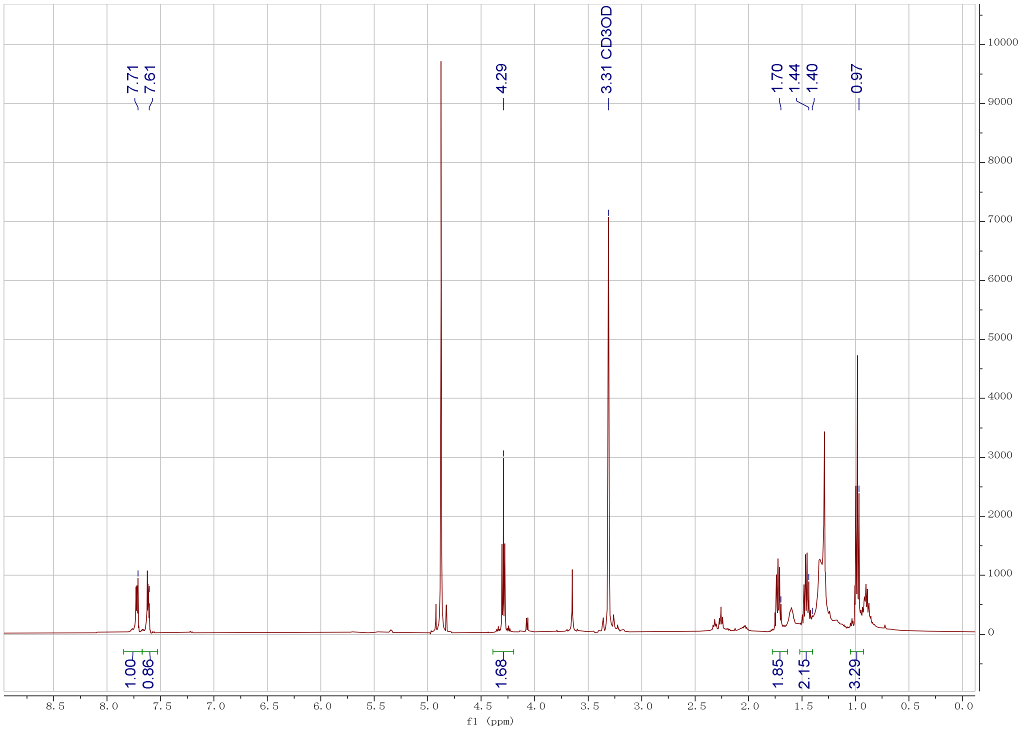

Figure S45. ^{1}H NMR spectrum of compound 21 (500 MHz, CD3OD)

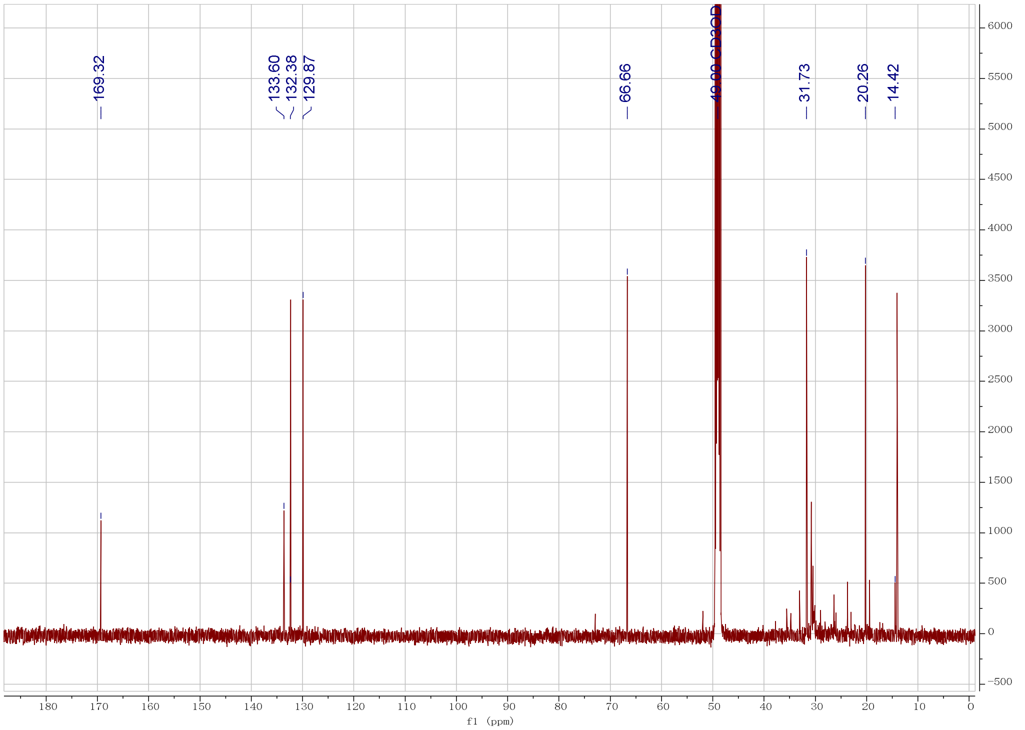

Figure S46. ^{13}C NMR spectrum of compound 21 (125 MHz, CD3OD)

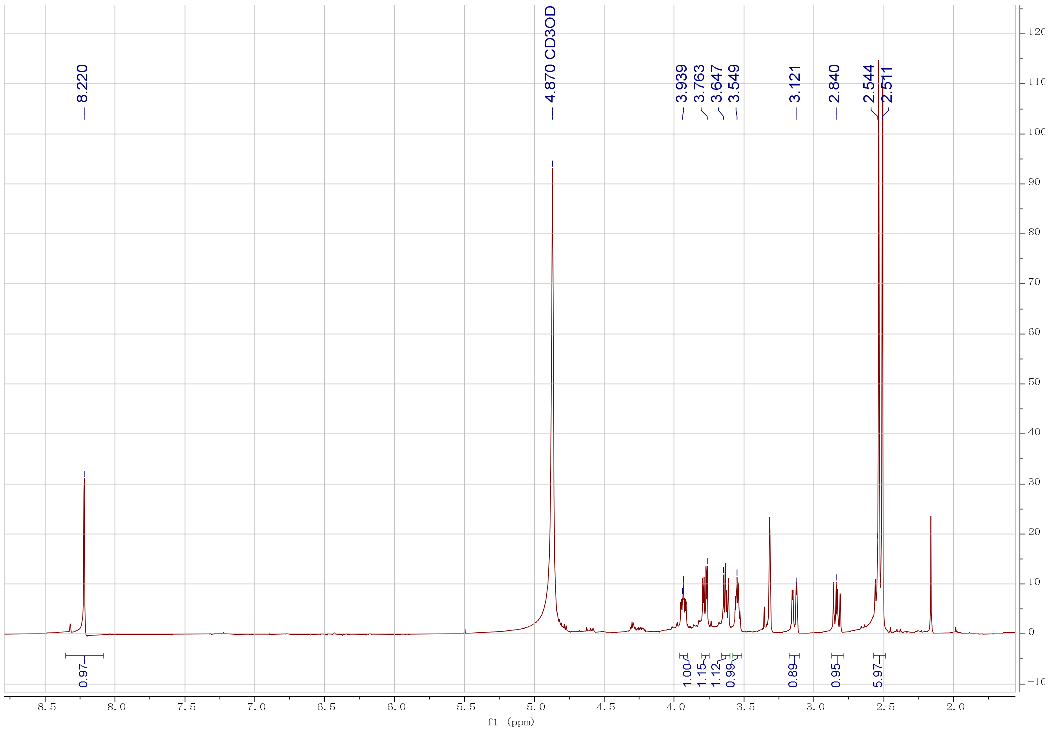

Figure S47. ^{1}H NMR spectrum of compound 34 (500 MHz, CD3OD)

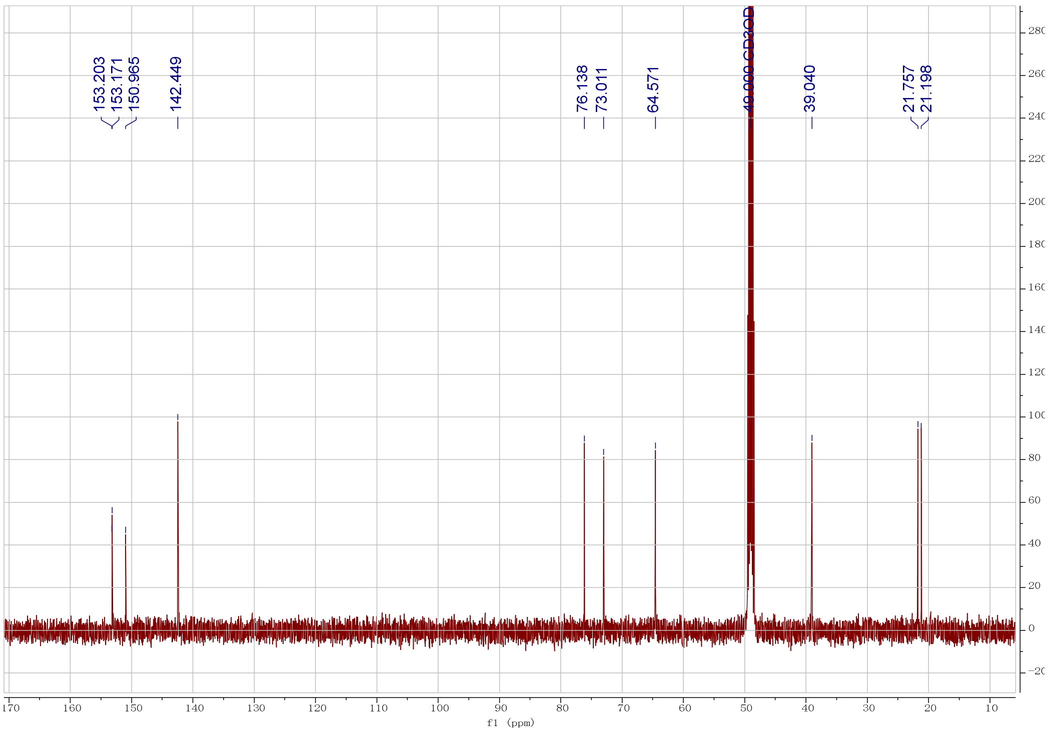

Figure S48. ^{13}C NMR spectrum of compound 34 (125 MHz, CD3OD)

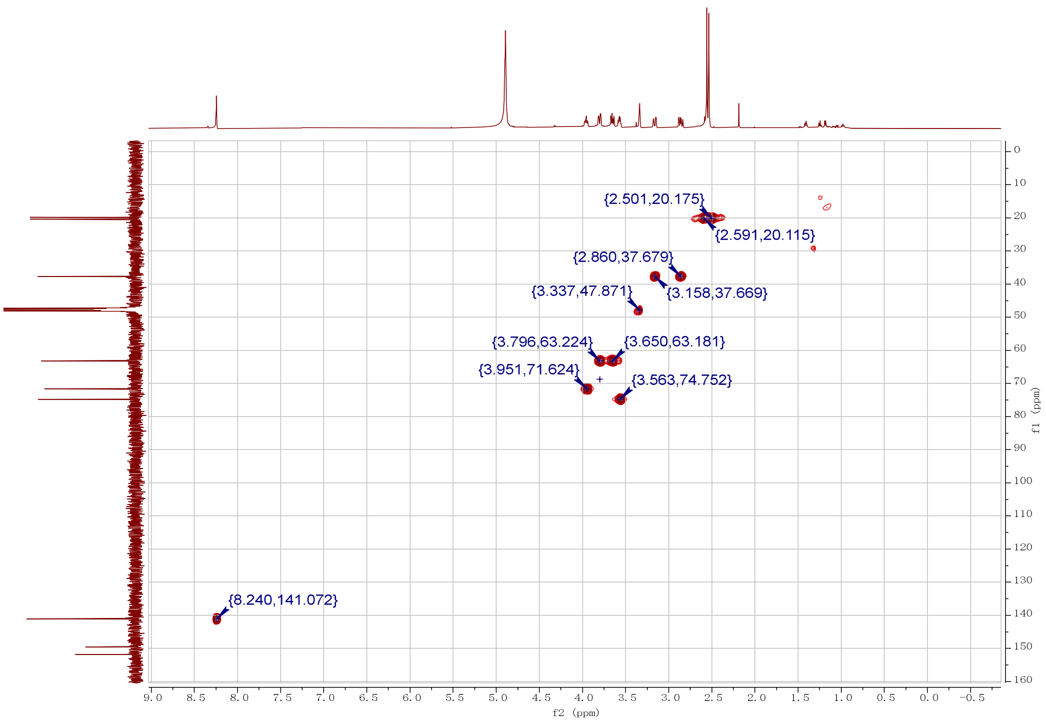

Figure S49. HSQC spectrum of compound 34 (500 MHz, CD3OD)

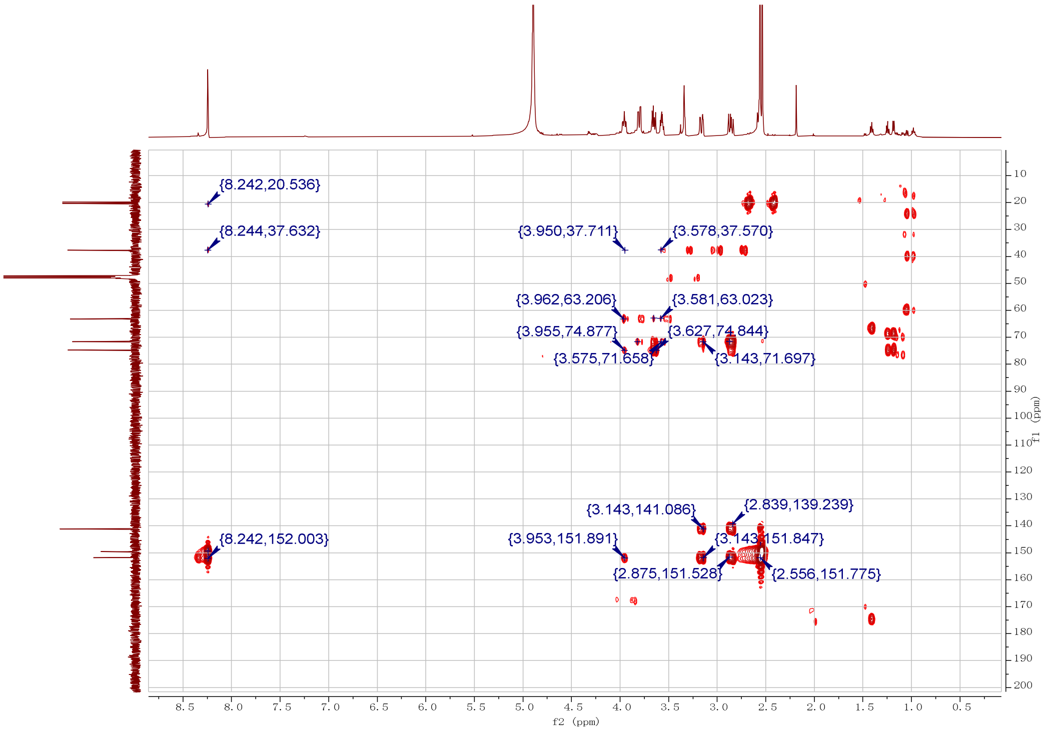

Figure S50. HMBC spectrum of compound 34(500 MHz, CD3OD)

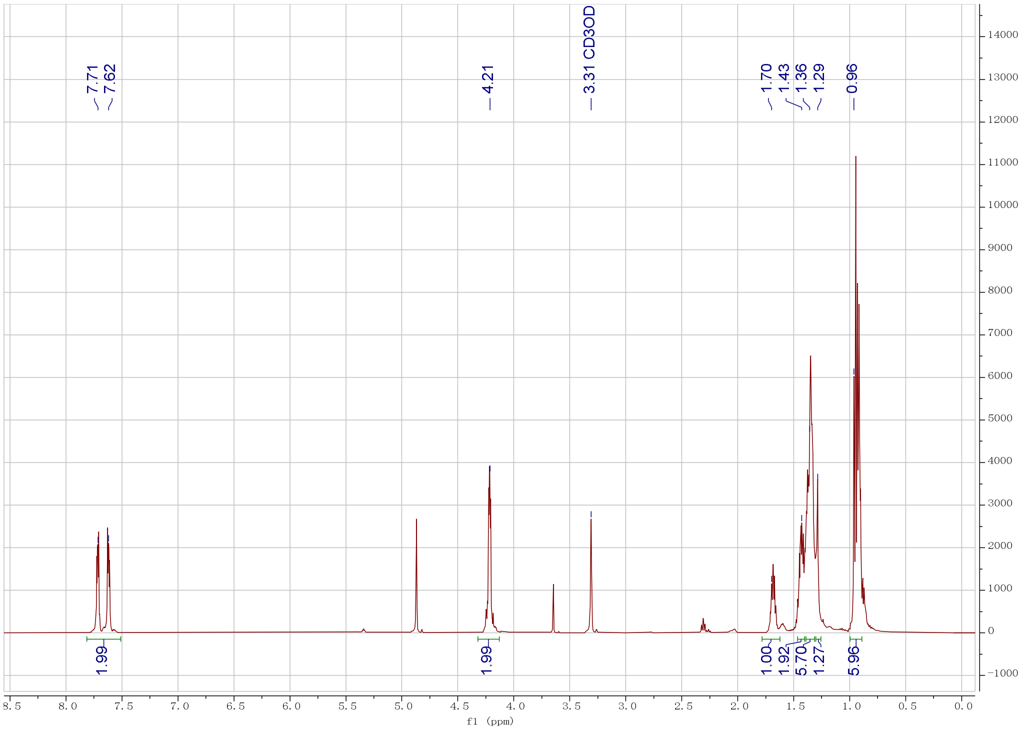

Figure S51. ^{1}H NMR spectrum of compound 35 (500 MHz, CD3OD)

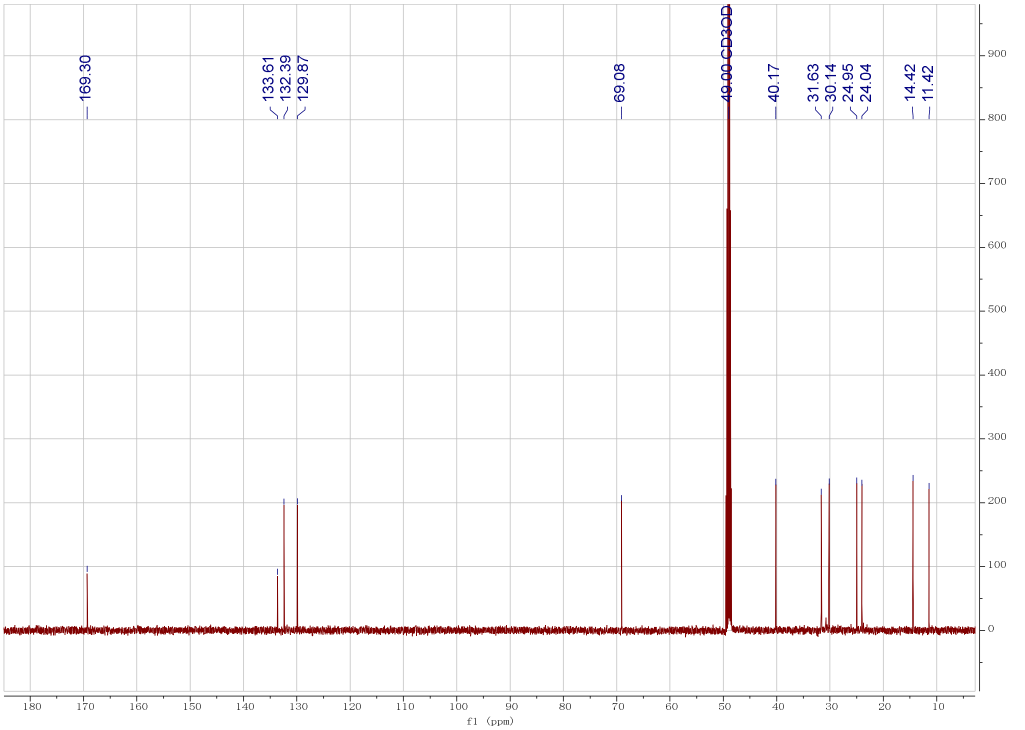

Figure S52. ^{13}C NMR spectrum of compound 35 (125 MHz, CD3OD)

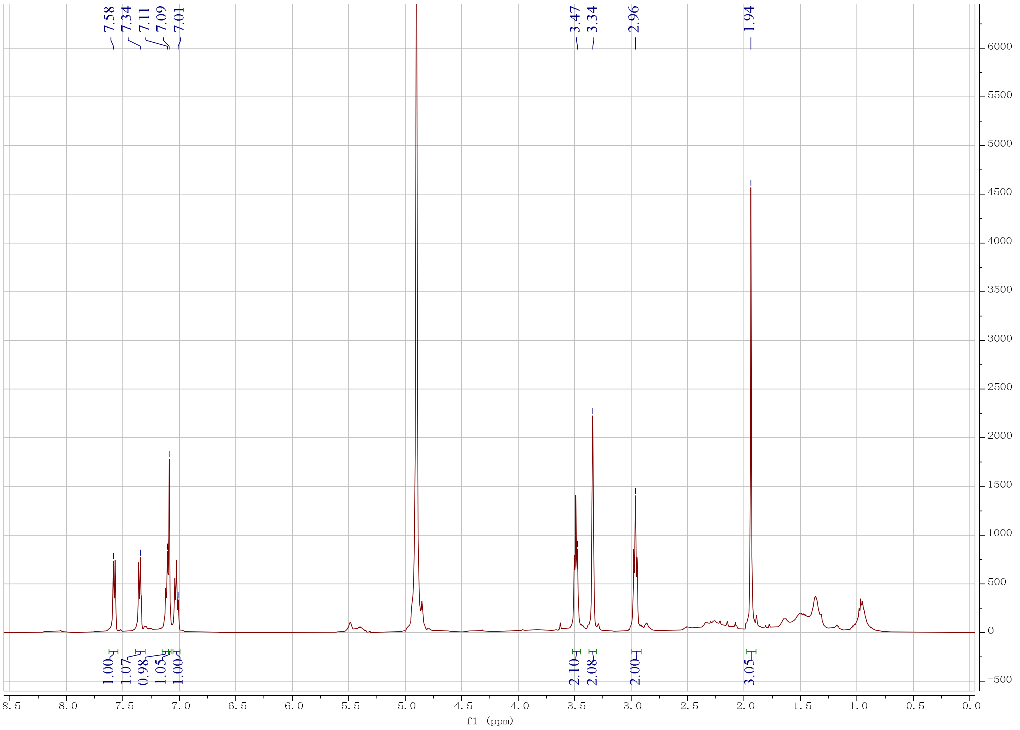

Figure S53. ^{1}H NMR spectrum of compound 36(500 MHz, CD3OD)

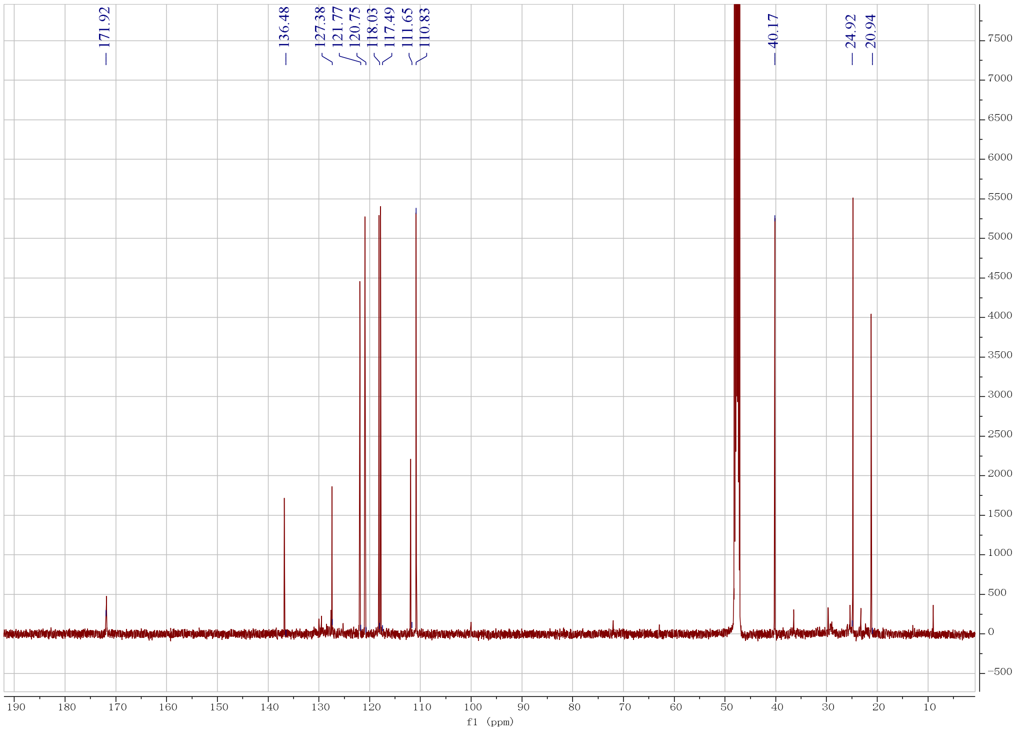

Figure S54. ^{13}C NMR spectrum of compound 36(125 MHz, CD3OD)

Table S1 Phenotypic characterization of strain TRM70308^T and its related strains

Characteristics	1	2	3	4	5	6
45℃	+	-	+	-	-	+
pH tolerance range	6-10	6-11	5-8	6-10	6-10	6-10
NaCl tolerance range	0-10%	0-10%	0-5%	0-10%	0-10%	0-10%
Raffinose	-	-	-	ND	+	+
Lactose	+	+	+	-	-	+
L–arabinose	+	-	+	-	-	-
Sucrose	+	-	-	-	+	ND
xylose	-	-	-	-	+	-
D-mannitol	+	-	-	-	-	+
glucose	+	-	+	+	-	+
meso-inositol	-	-	-	ND	+	-
D–fructose	+	-	+	-	+	+
L–rhamnose	+	-	+	+	+	+
Gelatin liquefaction	-	-	+	-	-	+
Urease	+	+	+	-	-	-
Nitrate reduction	+	-	+	-	+	-
Cellulose hydrolysis	+	+	-	-	+	-
Catalase production	-	+	+	+	ND	+
Starch hydrolysis	+	+	+	-	+	+

1. TRM70308^T, 2. *Streptomyces chumphonensis* KK1-2^T, 3. *Streptomyces lycii* TRM66187^T, 4. *Streptomyces gobiensis* 1-25^T, 5. *Streptomyces alkaliterrae* OF1^T, 6. *Streptomyces durbertensis* NEAU-S1GS20^T

Table S2. Key enzymes involved in the shikimic acid pathway in strain TRM70308

gene	name	location	size
4496	Shikimate dehydrogenase,SHD	5,468,884-5,469,711	828bp
4497	chorismate synthase	5469821-5,471,005	1185bp
4498	shikimate kinase	5,471,002-5,471,517	516bp
4499	3-dehydroquinate synthase	5,471,514-5,472,605	1092bp
4450	3-dehydroquinate dehydratase	5,472,602-5,473,072	471bp
1826	DAHP synthase	2,221,269-2,222,564bp	1296bp
3353	EPSP synthase	4,084,104-4,085,429	1326bp

Table S3. Antimicrobial activity diameter

Test Pathogens	Zone of Inhibition (mm)
Candida albicans ATCC 64550	26.1±0.36
Escherichia coli ATCC 25922	24.0±0.17
Staphylococcus aureusS.a ATCC 6538	9.43±0.40
